# Rational Design of Ni^3+^‐Rich Nickel Nitride Enables Paired Electrooxidation of Sterol and Hydrogen Evolution in a Flow Electrolyzer

**DOI:** 10.1002/advs.74527

**Published:** 2026-02-19

**Authors:** Suiqin Li, Jieyu Wang, Ge Feng, Jiahui He, Kai Li, Lihao Liu, Yuhang Wang, Zixian Jia, Yanfei Xu, Wenwu Zhong, Xing Zhong, Jianguo Wang

**Affiliations:** ^1^ Zhejiang Key Laboratory of Functional ionic Membrane Materials and Technology for Hydrogen Production Shaoxing University Shaoxing China; ^2^ State Key Laboratory of Green Chemical Synthesis and Conversion Zhejiang Key Laboratory of Surface and Interface Science and Engineering for Catalysts College of Chemical Engineering Zhejiang University of Technology Hangzhou China; ^3^ School of Environment and Natural Resources Zhejiang University of Science & Technology Hangzhou China

**Keywords:** electrocatalyst, electrooxidation, flow electrolyzer, high‐valent nickel, sterol

## Abstract

Electrooxidation of sterols is recognized as a promising approach for synthesizing value‐added steroid hormone pharmaceuticals. However, enhancing current density while delivering market‐required steroidal carbonyl products with high selectivity remains a significant challenge. Herein, we report a rational design of Ni^3+^‐rich active sites on nickel nitride (Ni_3_N) nanosheets via Pd‐induced electronic modulation, which is utilized for sterol oxidation coupled with hydrogen evolution reaction (HER). Experimental and theoretical analyses demonstrated that the strong electron‐withdrawing capability of Pd acts as an “electron pump,” accelerating the formation of high‐valent Ni^3+^ active centers by attracting electrons from Ni_3_N to Pd, facilitating the adsorption and activation of N‐(1‐hydroxy‐2,2,6,6‐tetramethylpiperidin‐4‐yl)acetamide (ACTH) during the oxidation process, thereby enhancing catalytic activity. The Pd‐Ni_3_N composite as a bifunctional electrocatalyst for producing carbonyl product **1b** (19‐Hydroxy‐4‐androstene‐3,17‐dione) and H_2_ concurrently at an industrial‐scale current of 5 A in a large flow electrolyzer, achieving productivities of 70.9 mmol/h for **1b** (with 99% selectivity) and 93.1 mmol/h for H_2_. This work provides a novel strategy for the electrosynthesis of valuable steroid intermediates alongside H_2_ production.

## Graphical abstract

The rational design of Ni^3+^‐rich active sites on nickel nitride nanosheets via Pd‐induced electronic modulation, which was employed in the electrooxidation of sterol coupled with H_2_ production in a large flow electrolyzer. This work offers a novel strategy for the design and synthesis of highly efficient electrocatalysts and electrolyzers toward the electrosynthesis of valuable steroid intermediates coupled H_2_ production.

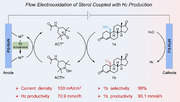

## 1 Introduction

Designing and constructing highly efficient active sites is a key challenge in addressing reaction kinetic bottlenecks in electrocatalytic oxidation reactions [1, 2, 3, 4, 5]. Notably, electrochemically generated high valence nickel (Ni^3+^) species, characterized by their unique e_g_ orbital configuration and abundant 3d electrons, exhibit remarkable catalytic efficacy in various oxidation reactions [6, 7, 8, 9]. The reaction mechanism primarily involves Ni^3+^ acting as a strong electrophile to selectively attack electron‐rich functional groups, activating C─H and O─H bonds via a proton‐coupled electron transfer mechanism, and subsequently oxidizing the molecules into the corresponding aldehyde or carboxylic acid products [10, 11, 12]. Thus, the comprehensive understanding of the formation pathway of Ni^3+^ and the development of effective strategies to control its electronic structure are of pivotal significance for the development of high‐performance electrocatalytic systems [13, 14, 15]. Nevertheless, the formation and stabilization of Ni^3+^ in conventional nickel‐based materials (e.g., Ni(OH)_2_/NiOOH) face considerable challenges [16]. The energy barrier associated with the conversion of Ni^2+^ to Ni^3+^ restricts the availability of active sites. Additionally, structural reconstruction and the dissolution of active species commonly occur during sustained anodic polarization, severely restricting their catalytic efficiency and stability.

Transition metal nitrides, particularly Ni_3_N, offer a promising pathway for promoting Ni^3+^ formation owing to their noble metal‐like electronic properties, high intrinsic conductivity, and tunable nitrogen vacancy structures [17, 18, 19, 20, 21]. The robust interaction between Ni 3d and N 2p orbitals within Ni_3_N plays a pivotal role in stabilizing high‐valent nickel species and inducing the formation of catalytically active Ni^3+^ sites via a dynamic surface restructuring process [22, 23]. For instance, vanadium atoms modulate the charge distribution and bonding strength of Ni–N bonds, promoting nitrogen vacancy formation and enhancing the adsorption of OH* species, thereby significantly accelerating the surface reconstruction of Ni_3_N into catalytically active NiOOH phases [24]. Nevertheless, single‑component Ni_3_N materials suffer from limited intrinsic Ni^3+^ content, relatively high surface‑reconstruction barriers, and structural instability in strongly alkaline media. To address these issues, introducing palladium (Pd) with a high work function to construct a heterogeneous interface has emerged as an effective strategy. According to the d‐band center theory, the formation of Pd^δ−^–Ni^δ+^ polarized units at the Pd and Ni_3_N interface generates an “electron‑pump” effect [25]. The strong electron‐withdrawing capability of Pd elevates the energy level of Ni 2p orbitals, lowering the oxidation overpotential for the Ni^2+^ to Ni^3+^ conversion and enabling directional enrichment of Ni^3+^. Meanwhile, the formed Pd–Ni–N coordination structure enhances the stability of Ni─N bonds, effectively suppressing hydrolytic deactivation in alkaline environments [26, 27, 28, 29]. Additionally, Pd sites optimize the adsorption of hydrogen intermediates, and their synergistic interaction with Ni─N sites endows the composite with superior hydrogen evolution activity, establishing a highly efficient bifunctional catalytic mechanism [30, 31].

In recent years, replacing the kinetically sluggish oxygen evolution reaction (OER) with the thermodynamically more favorable electrooxidation of small‐molecule organics (alcohols, amines, alkenes, etc.) has been validated as an effective approach to achieve high‑value products [32, 33, 34, 35, 36, 37, 38]. As a representative example, the sterols oxidation to produce corresponding steroid carbonyl products is a critical step in the manufacture of steroid‐based pharmaceuticals [39, 40, 41, 42]. Existing industrial processes rely on chromic acid as the oxidant, which is associated with severe environmental pollution and high energy consumption [43]. A promising strategy is integrating the selective electrooxidation of sterols with the HER, which produces both steroid carbonyl chemicals and hydrogen with high energy density due to its mild and controllable operational conditions. However, the industrial‐scale application of the sterol oxidation process remains challenging due to the intrinsic molecular complexity of sterols, including their substantial steric hindrance, poor aqueous solubility in alkaline electrolytes, and the limitations of conventional batch reactor configurations. These factors collectively contribute to rapid active site deactivation, severe mass transfer limitations, and sluggish reaction kinetics. Accordingly, it is crucial to develop highly efficient electrocatalysts and electrolyzers for achieving the coupling of sterols oxidation and HER.

Hence, we designed a flow electrolysis system based on 3D porous graphite felt (GF), wherein vertically aligned Pd‐Ni_3_N nanosheet arrays were constructed on the electrode surface. This configuration utilizes forced convection to reduce diffusion layer thickness while enabling efficient exposure and utilization of active sites. The combination of in situ electrochemical experiments and theoretical calculations elucidated the electronic mechanism of Pd‐induced Ni^3+^ active centers enrichment, and the adsorption‐regeneration kinetics of the 4‐acetamido‐TEMPO (ACT) mediator at Ni^3+^ sites. Furthermore, optimizing the matching of anodic and cathodic reactions in the flow electrolyzer enabled synergistic and efficient sterols oxidation and HER at an industrial‐scale current of 5 A, achieving productivities of 70.9 and 93.1 mmol/h for steroidal carbonyl product and H_2_, respectively.

## 2 Results and Discussion

### 2.1 Synthesis and Characterization of Pd‐Ni_3_N/GF

**FIGURE 1:**
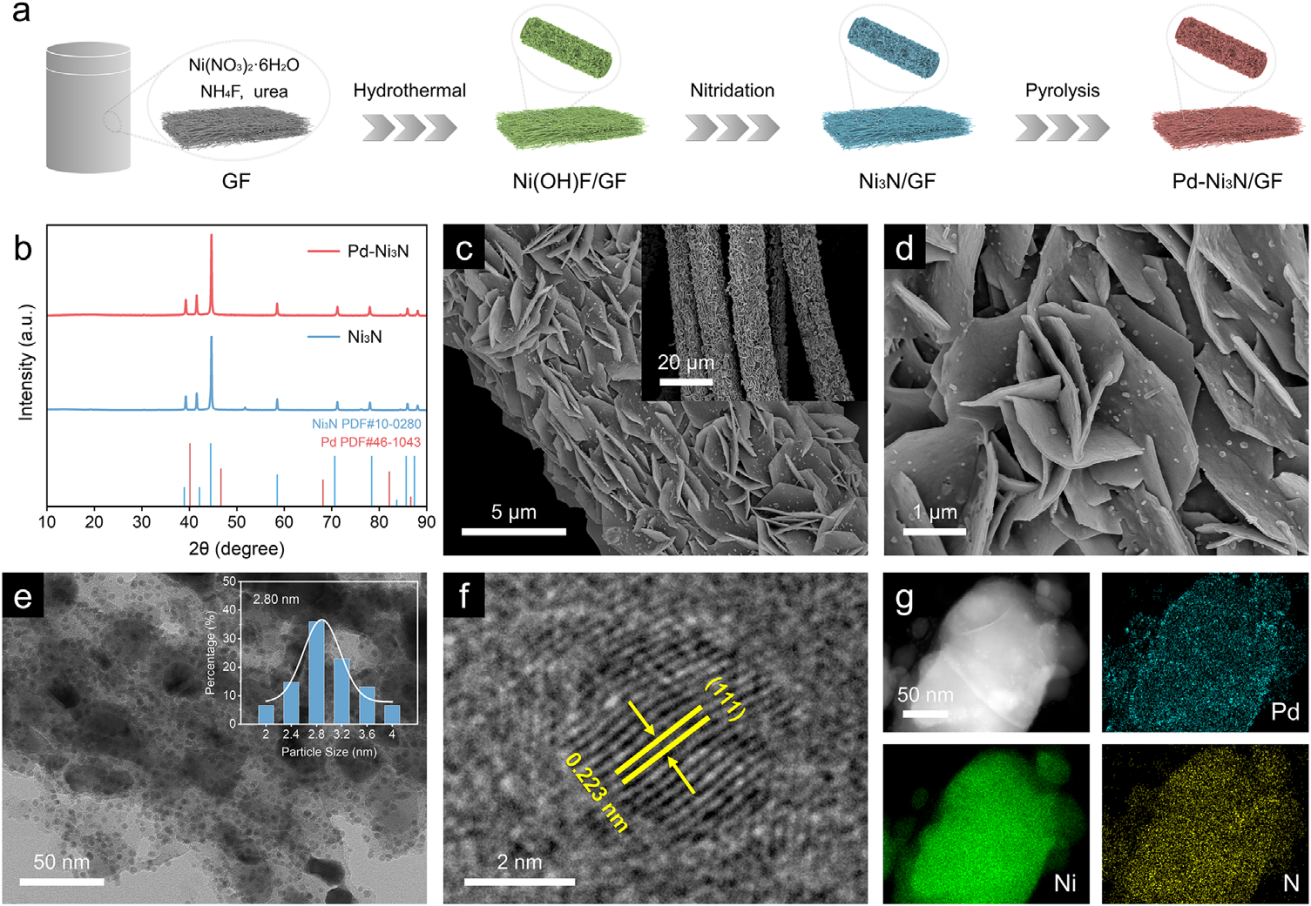
(a) Schematic of the preparation method of Pd‐Ni_3_N/GF. (b) XRD patterns of Pd‐Ni_3_N and Ni_3_N. (c‐d) SEM images morphology of Pd‐Ni_3_N/GF, inset of (c) is the corresponding low‐magnification SEM image. (e) TEM, (f) HRTEM, and (g) Elemental mapping of Pd‐Ni_3_N/GF, inset of (e) is the corresponding Pd particle size distribution plot.

The Pd‐Ni_3_N/GF electrode was fabricated through a three‐step process, as illustrated schematically in Figure 1a. The process commenced with the hydrothermal synthesis of vertically aligned Ni(OH)F precursor nanosheets on a graphite felt substrate, utilizing nickel nitrate hexahydrate as the metallic precursor in conjunction with urea and ammonium fluoride as coordinating agents. This was followed by a nitridation treatment under ammonia gas atmosphere, which effectively converted the hydroxide fluoride precursor into Ni_3_N/GF. Finally, Pd nanoparticles were deposited onto the nanostructured surface through controlled thermal decomposition, obtaining the desired Pd‐Ni_3_N/GF electrocatalyst. The loading of Pd was identified as 3.43 wt.% by inductively coupled plasma mass spectrometry (ICP‐MS) analysis. To eliminate any potential interference from the substrate GF during the structural characterisation process, Pd‐Ni_3_N powder catalysts were synthesized using the same method for X‐ray diffraction (XRD) phase analysis. As shown in Figure 1b, the XRD pattern of Ni_3_N exhibites characteristic peaks at 39.2°, 41.5°, 44.7°, and 58.4°, and 71.2°, corresponding to the (110), (002), (111), (112), and (300) planes of hexagonal Ni_3_N phase (JCPDS No. 10–0280), respectively [44, 45]. However, no characteristic diffraction peaks of metallic Pd were detected in the XRD pattern after Pd incorporation, which is primarily attributed to the limited detection sensitivity of XRD for low‐concentration dopants (<5 wt.%), a conclusion corroborated by ICP‐MS results.

Scanning electron microscopy (SEM) analysis reveals that Pd‐Ni_3_N nanosheets were uniformly grown on the GF surface (Figure 1c; Figure S1), exhibiting a pronounced 3D rough morphology (Figure 1d). This hierarchical architecture facilitates the exposure of abundant active sites and enhances solid‐liquid interfacial mass transfer efficiency, thereby improving electrocatalytic performance. Figures S2 and S3 show that Ni(OH)F/GF and Ni_3_N/GF have similar nanosheet morphologies as Pd‐Ni_3_N/GF assembled on GF densely. Transmission electron microscopy (TEM) analysis demonstrates that pristine Ni_3_N nanosheets possess relatively smooth surface structures (Figure S4a), while the Pd‐modified sample shows uniform distribution of metallic nanoparticles across the nanosheet surfaces (Figure 1e). Statistical analysis of the particle size distribution confirms monodisperse Pd nanoparticles with a diameter of approximately 2.8 nm, presented in the inset of Figure 1e. Moreover, the high‐resolution TEM (HRTEM) investigations show lattice spacings of 0.204 nm within the Ni_3_N (Figure S4b), corresponding to the (111) plane of Ni_3_N [44]. The loaded Pd nanoparticles display a clear interplanar distance of 0.223 nm belonging to the (111) plane of the metallic Pd (Figure 1f), which is consistent with the XRD result. The elemental mapping analysis reveals a uniform distribution of Pd, Ni, and N elements throughout the whole nanosheet (Figure 1g), further supporting the successful loading of Pd into the Ni_3_N.

**FIGURE 2:**
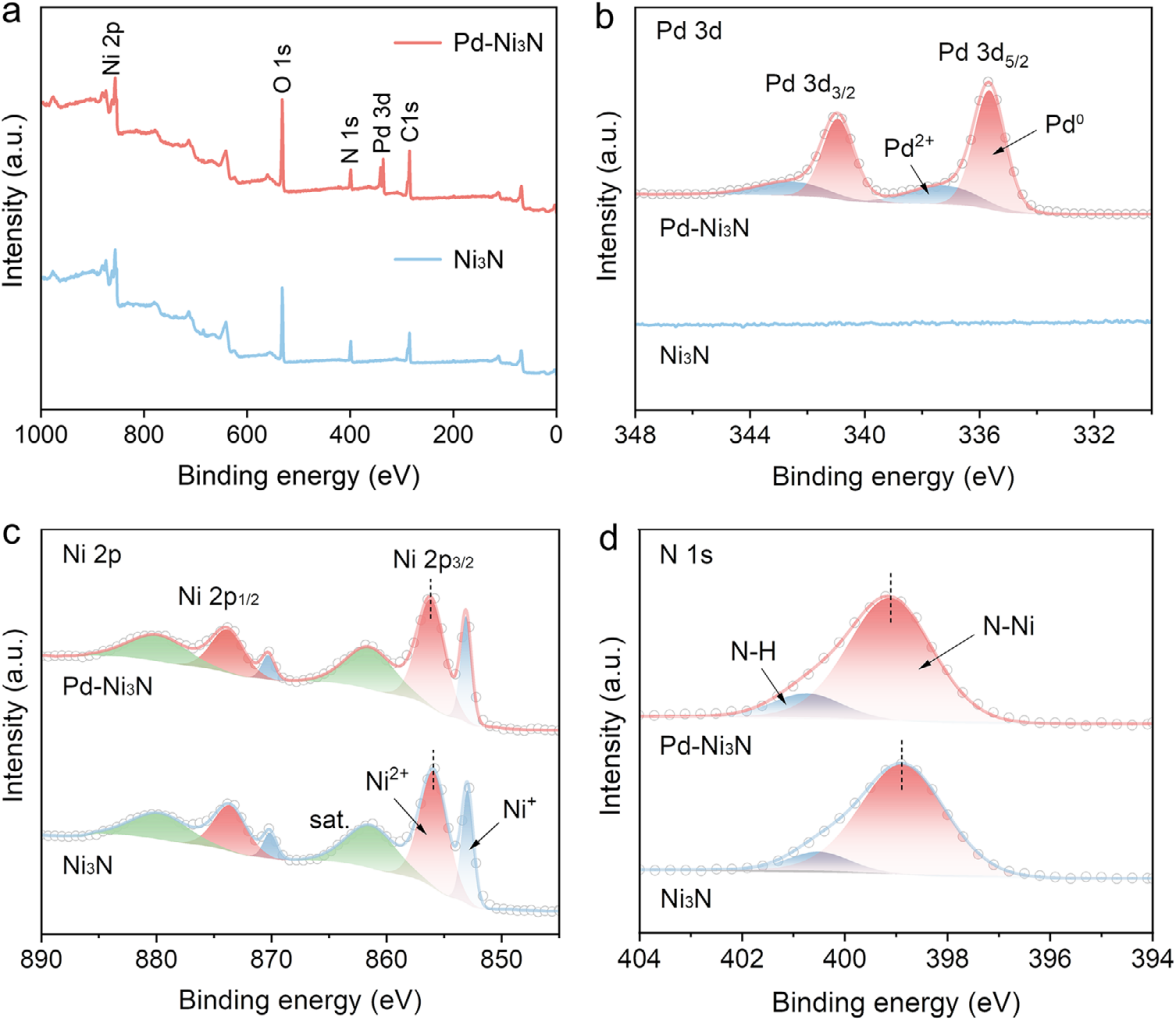
(a) XPS survey spectra of Pd‐Ni_3_N and Ni_3_N. XPS high‐resolution spectra of (b) Pd 3d, (c) Ni 2p and, (d) N 1s for Pd‐Ni_3_N and Ni_3_N.

The surface chemical valence and electronic structure of Pd‐Ni_3_N catalysts were systematically analyzed by X‐ray photoelectron spectroscopy (XPS). Figure 2a displays the presence of Pd, Ni, and N in the survey spectrum, which is consistent with the results of the elemental mapping discussed above, while Pd signals are absent in Ni_3_N. The high‐resolution spectrum of Pd 3d manifests a predominant Pd^0^ signal, accompanied by a marginal Pd^2+^ signal (Figure 2b), confirming the successful loading of the Pd into Ni_3_N. The deconvolution of the Ni 2p_3/2_ envelope for Pd‐Ni_3_N demonstrated three well‐resolved peaks at 853.0, 856.1, and 861.4 eV (Figure 2c), which were assigned to the Ni─N bond, Ni─O bond, and satellite peak from the shake‐up excitation, respectively [9, 18, 36, 46]. Noteworthily, the binding energies of Ni in Pd‐Ni_3_N exhibit a positive shift compared with that of pure Ni_3_N, suggesting that the introduction of Pd facilitates electron transfer from Ni to Pd, accelerating the formation of high‐valent nickel ions. The N 1s spectrum can be deconvoluted into two peaks at 399.1 and 400.7 eV (Figure 2d), which are attributed to N‐Ni species and N‐H species of Pd‐Ni_3_N, respectively [46]. The peak for N 1s of Pd‐Ni_3_N also positively shifts toward higher binding energy compared to Ni_3_N. These data indicate that beneficial electron transfer and charge density redistribution occur at the Pd‐Ni_3_N interface. The XPS spectra further prove that Pd‐Ni_3_N nanosheets were successfully fabricated.

### 2.2 Electrocatalytic Performance of Pd‐Ni_3_N/GF for 1a Electrooxidation

**FIGURE 3:**
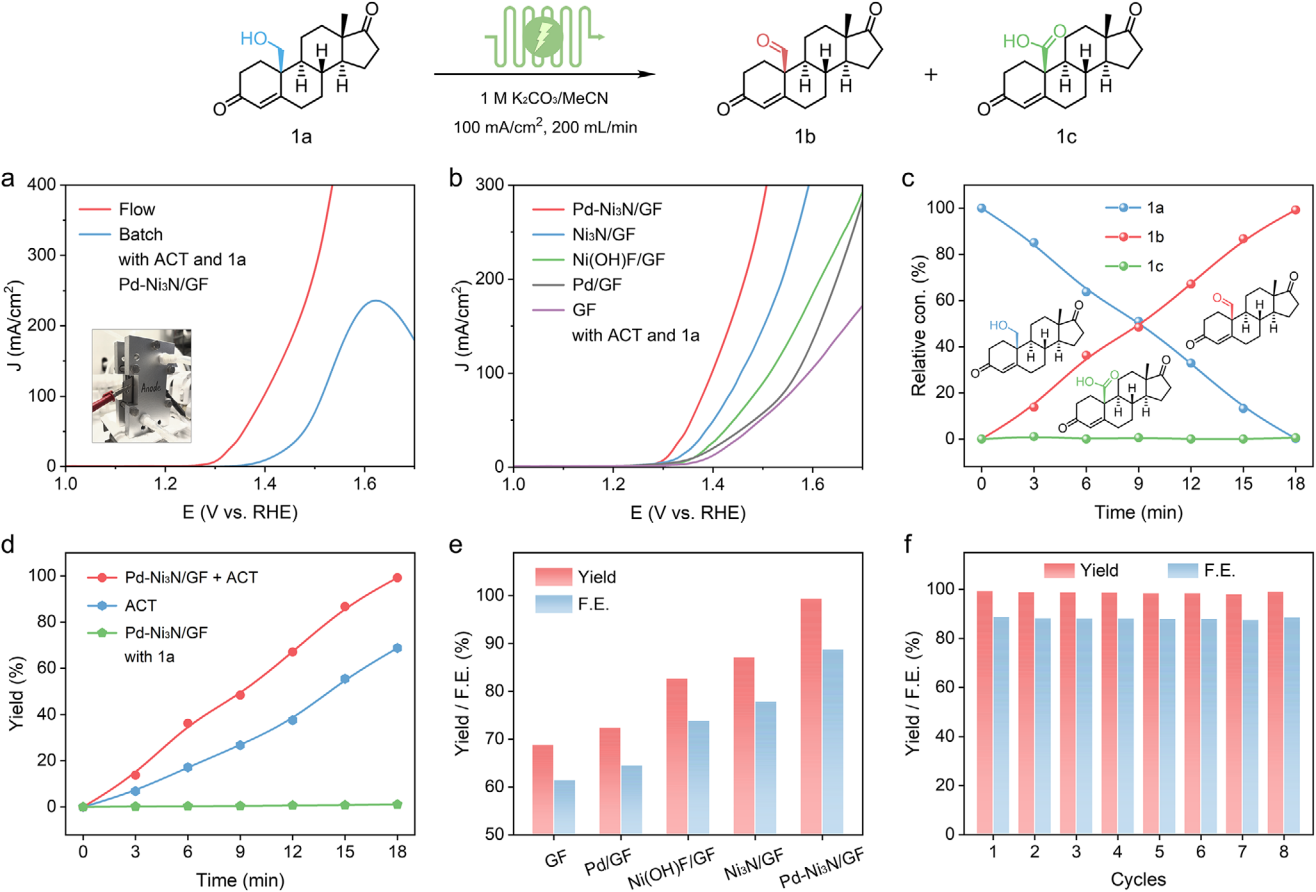
(a) LSV curves of Pd‐Ni_3_N/GF in 1 m K_2_CO_3_ electrolyte in the presence of **1a** and ACT in the batch reactor and flow electrolyzer, inset of is the diagram of the flow electrolyzer. (b) LSV curves of Pd‐Ni_3_N/GF, Ni_3_N/GF, Ni(OH)F/GF, Pd/GF, and GF in 1 m K_2_CO_3_ in the presence of **1a** and ACT in a flow electrolyzer. (c) Relative concentration (Relative Con.) changes (%) of **1a** and its oxidation products (**1b**, **1c**) during the **1a** electrooxidation in the flow electrolyzer at a current density of 100 mA/cm^2^. (d) Yield (%) of products during **1a** electrooxidation in a flow electrolyzer for Pd‐Ni_3_N/GF, ACT, and Pd‐Ni_3_N/GF + ACT. (e) Yield (%) and F.E. (%) of products during **1a** electrooxidation in a flow electrolyzer by different anode electrocatalysts of Pd‐Ni_3_N/GF, Ni_3_N/GF, Ni(OH)F/GF, Pd/GF, and GF. (f) Yield (%) and F.E. (%) of product under 8 continuous electrolysis cycles.

The electrocatalytic performance of the as‐prepared Pd‐Ni_3_N/GF for the electrooxidation of sterol was assessed in 1 m K_2_CO_3_ with or without 50 mm **1a** (19‐hydroxyandrost‐4‐ene‐3,17‐dione). Initially, the electrocatalytic performance of the Pd‐Ni_3_N/GF, Ni_3_N/GF, and Ni(OH)F/GF electrocatalysts was assessed in a batch reactor employing a standard three‐electrode system in 1 m K_2_CO_3_ electrolyte (Figure S5). In this system, the Hg/HgO and Pt sheets were used as the reference and counter electrodes, respectively. Additionally, we incorporated 4‐acetamido‐2,2,6,6‐tetramethyl‐1‐piperidine‐N‐oxyl (ACT) as a mediator [47, 48, 49, 50], which has been demonstrated to exhibit outstanding alcohol oxidation performance when coupled with heterogeneous catalysts [16, 40, 51]. As illustrated in Figure S6, an oxidation peak at ≈1.40 V vs. RHE was observed because of the oxidation of Ni^2+^‐OH to Ni^3+^‐OOH, which is the key active site for the electrocatalytic oxidation of alcohols [13, 28, 52]. After adding **1a** and ACT in 1 m K_2_CO_3_ electrolyte, the LSV curve of the Pd‐Ni_3_N/GF electrocatalyst displays an obvious current response within the potential window, indicating that the Pd‐Ni_3_N/GF catalyst is sensitive to ACT‐mediated electrocatalytic oxidation of **1a**. Subsequently, **1a** oxidation performance was further investigated by using a flow electrolyzer (Figure S7), which significantly improved the mass transfer efficiency and effectively reduced the concentration polarization compared to a conventional batch reactor, thus offering the potential to increase the current density [53, 54]. As illustrated in Figure 3a, the Pd‐Ni_3_N/GF electrocatalyst shows a huge current increase for the **1a** oxidation in a flow electrolyzer, achieving a large current density of 279 mA/cm^2^ at 1.50 V vs. RHE, which is more than 3.7‐fold higher than that of the batch reactor (76 mA/cm^2^). Such a high current density is expected to shorten the reaction time required for **1a** oxidation and increase the space‐time yield of the target product **1b**. Additionally, an integrated Pd‐Ni_3_N/GF and ACT electrocatalyst exhibited significantly higher **1a** oxidation performance than Pd‐Ni_3_N/GF or ACT alone (Figure S8), suggesting a synergistic catalytic effect between Pd‐Ni_3_N/GF and ACT for the **1a** oxidation process. The 5% Pd‐Ni_3_N/GF displays the excellent **1a** oxidation activity with the higher oxidation current density (Figure S9), thereby establishing itself as the optimal homogeneous electrocatalyst for the subsequent discussion. Besides, the **1a** oxidation performance of different electrode materials is shown in Figure 3b, where the Pd‐Ni_3_N/GF electrode exhibits the lowest potential for **1a** oxidation and the most significant current response under the same reaction conditions compared to the Ni_3_N/GF, Ni(OH)F/GF, Pd/GF, and GF electrodes, highlighting the pivotal role of Pd in enhancing **1a** oxidation activity. The corresponding Tafel slopes were calculated to evaluate the kinetics feature for ACT‐mediated electrocatalytic oxidation of **1a**. Figure S10 showed the Tafel slope value of Pd‐Ni_3_N/GF is 99 mV/dec, smaller than that of Ni_3_N/GF (132 mV/dec), Ni(OH)F/GF (156 mV/dec), Pd/GF (220 mV/dec), and GF (201 mV/dec), implying a faster electron‐transfer rate and superior catalytic kinetics for **1a** oxidation. The electrochemical surface areas (ECSA) were compared by estimating their electrochemical double‐layer capacitance (C_dl_) in the non‐faradaic regions using cyclic voltammetry (CV) curves at different scan rates (Figure S11). The C_dl_ value of Pd‐Ni_3_N/GF (4.72 mF/cm^2^) was much higher than that of Ni_3_N/GF (2.87 mF/cm^2^), evidencing increased catalytic sites after Pd loading into Ni_3_N (Figure S12). These results, combined with their uniform nanosheet‐like structure, verify that more active sites could be exposed by introducing Pd, which can significantly enhance the catalytic activity of Pd‐Ni_3_N/GF toward ACT‐mediated electrocatalytic oxidation of **1a**.

To identify and quantify the oxidation products, Pd‐Ni_3_N/GF‐catalyzed **1a** electrooxidation was carried out at a constant current of 1 A (current density of 100 mA/cm^2^) in a flow electrolyzer, and then the reactants/products were detected by high‐performance liquid chromatography (HPLC). Theoretically, owing to the complex two‐electron transfer, a charge of 965 C is required to convert 5 mmol **1a** (50 mm **1a** in 100 mL 1 m K_2_CO_3_ electrolyte) into **1b** completely. During the **1a** oxidation process, the anode potential remained relatively stable at approximately 1.52 V vs. RHE, followed the potential underwent a gradual increase as the reaction approached completion (Figure S13). Concurrently, the accumulated charge quantity progressively reached 1080 C over time. After 18 min, **1a** almost disappears in the system (Figure 3c), while the **1b** content increases to the maximum, and the by‐product acid **1c** was very low (<1%), elucidating the complete conversion from **1a** to **1b**. As illustrated in Figure 3d, the integration of Pd‐Ni_3_N/GF and ACT as co‐catalysts achieved a remarkable yield of 98% within 18 min, whereas the sole use of ACT resulted in a significantly lower yield (68%), indicating limited catalytic efficiency despite its inherent activity. Pd‐Ni_3_N/GF alone exhibited negligible reactivity, with a yield of merely 2% (Figure S14), suggesting minimal interaction between **1a** and the Pd‐Ni_3_N/GF electrode surface. These results demonstrated a significant synergistic catalytic interaction between Pd‐Ni_3_N/GF and ACT, where ACT likely serves as the primary active site for the electrooxidation of sterol **1a**, while the Pd‐Ni_3_N/GF surface facilitates ACT adsorption and activation, thereby enhancing the overall catalytic performance. Notably, the Pd‐Ni_3_N/GF exhibits impressive ACT‐mediated electrooxidation performance at high current density with high **1b** selectivity of 99%, yield of 98%, and F.E. of 88%, outperforming that of the Ni_3_N/GF, Ni(OH)F/GF, and Pd/GF (Figure 3e). This result unequivocally demonstrates that the high activity for sterol oxidation is not attributable to the intrinsic catalytic property of Pd, but rather to the Pd‐induced electronic modulation of the Ni_3_N support, which enriches the surface Ni^3+^ active sites crucial for ACT adsorption and activation. The constant‐current electrolysis and stability tests demonstrated that the amount of Pd loaded has a significant impact on the selectivity of the products and the durability of the catalyst (Figure S15), further confirming the existence of an “optimal loading window” of approximately 5% Pd. Additionally, when Pd‐Ni_3_N/GF was employed as the anode, it was observed that altering cathode materials (e.g., Pd‐Ni_3_N/GF, GF, or nickel foam (NF)) significantly modulated the **1a** oxidation performance (Figure S16). This phenomenon highlights the pivotal role of cathode catalyst selection in enhancing the anode‐driven **1a** oxidation kinetics, suggesting that high‐performance cathode catalysts can synergistically promote the electrochemical activity of the anode reaction. Besides, the difference in redox potential between TEMPO and ACT results in longer reaction times and lower F.E. (Figure S17). Moreover, the **1a** electrooxidation performance exhibited a significant response to alterations in both the dosage of ACT and the concentration of K_2_CO_3_ (Figures S18 and S19). In addition, the ACT‐mediated electrooxidation of **1a** over the Pd‐Ni_3_N/GF electrode was carried out in a batch reactor. As shown in Figure S20, the yield reached only 35% after 600 min of electrolysis, which was attributed to severe concentration polarization within the batch reactor system, resulting in sluggish reaction kinetics and prolonged reaction duration. Furthermore, the 8 continuous electrolysis cycles (at a constant current of 1 A) were further evaluated to explore the stability and durability of the ACT‐mediated electrooxidation of **1a** over the Pd‐Ni_3_N/GF electrode. Impressively, it retains a high **1b** yield (>96%) and F.E. (>86%), showcasing outstanding stability of Pd‐Ni_3_N/GF (Figure 3f). These results demonstrated that the Pd‐Ni_3_N/GF is a highly active, selective, and stable catalyst for the ACT‐mediated electrooxidation of **1a** into **1b**.

### 2.3 Electrocatalytic Performance of Pd‐Ni_3_N/GF for 1a Electrooxidation and HER in a Large Flow Electrolyzer

**FIGURE 4:**
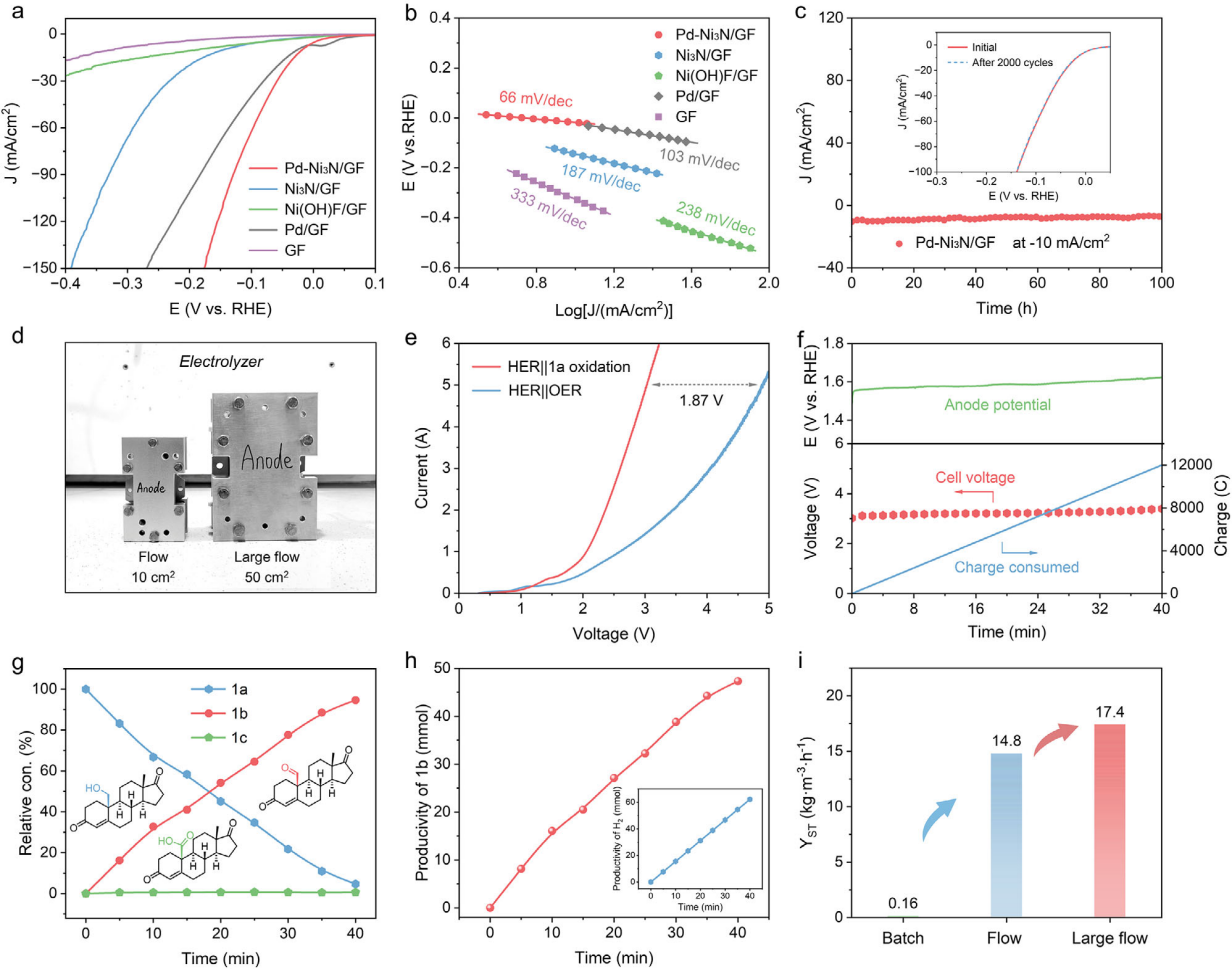
(a) HER polarization curves of Pd‐Ni_3_N/GF, Ni_3_N/GF, Ni(OH)F/GF, Pd/GF, and GF in 1 m KOH. (b) The corresponding Tafel slopes of Pd‐Ni_3_N/GF, Ni_3_N/GF, Ni(OH)F/GF, Pd/GF, and GF. (c) The chronoamperometry response of Pd‐Ni_3_N/GF at J = ‐10 mA/cm^2^ for HER, the inset is LSV curves of Pd‐Ni_3_N/GF before and after 2000 CV cycles in 1 m KOH. (d) Comparison of the flow electrolyzer and the large flow electrolyzer. (e) LSV curves comparison for electrolytic water and **1a** oxidation (**1a**, 15 g) in the large flow electrolyzer. (f) Cell voltage, anode potential, and charge passed for the Pd‐Ni_3_N/ACT‐mediated electrocatalytic oxidation of **1a** in the large flow electrolyzer. (g) Relative con. (%) of **1a**, **1b**, and **1c**. (h) Productivity of **1b** and H_2_ in the large flow electrolyzer at 5 A (100 mA/cm^2^). (i) Space‐time yield of **1a** electrooxidation for Pd‐Ni_3_N/GF in the batch reactor, flow electrolyzer, and large flow electrolyzer.

In addition to the remarkable **1a** electrooxidation activity, the electrocatalytic HER performance of the Pd‐Ni_3_N/GF electrode was also conducted by using a three‐electrode system in 1 m KOH solution. As the LSV curves shown in Figure 4a, Pd‐Ni_3_N/GF exhibits superior HER activity with a low overpotential of 18 mV at a current density of ─10 mA/cm^2^, which is greatly lower than those of Ni_3_N/GF (145 mV), Ni(OH)F/GF (185 mV), Pd/GF (28 mV) and GF (322 mV), underscores a synergistic interfacial effect. Remarkably, the Pd‐Ni_3_N/GF delivers a Pt‐like HER activity with an overpotential of 139 mV at ─100 mA/cm^2^, outperforms the contrast samples at large current density (Figure S21 and Table S1), ascribing to the sheet‐like nanostructure with highly accessible active sites and easy gas bubble release. The corresponding Tafel slope of Pd‐Ni_3_N/GF is 66 mV/dec (Figure 4b), significantly smaller than those of Ni_3_N/GF (187 mV/dec), Ni(OH)F/GF (238 mV/dec), Pd/GF (197 mV) and GF (333 mV/dec), revealing a faster HER kinetics of Pd‐Ni_3_N/GF. The enhanced kinetics are attributed to the Pd‐Ni_3_N interface, where the electron‐withdrawing Pd acts as an “electron pump,” optimizing the hydrogen adsorption energy on adjacent Ni sites and facilitating charge transfer, rather than merely providing additional Pd active sites. The excellent kinetic property of Pd‐Ni_3_N/GF was further validated by electrochemical impedance spectroscopy (EIS). In Figure S22, the Nyquist plots indicated that the Pd‐Ni_3_N/GF possesses the smallest charge transfer resistance (R_ct_) compared to Ni_3_N/GF, implying the faster electron‐transfer for the HER. Additionally, in order to investigate the stability of Pd‐Ni_3_N/GF toward HER (Figure 4c), the LSV curves obtained after 2000 continuous cycles almost overlapped with the initial LSV curves (inset of Figure 4c), and the Pd‐Ni_3_N/GF maintained a stable polarization current without significant loss in the long‐term controlled potential electrolysis test over 100 h (constant current density of ‐10 mA/cm^2^), demonstrating the outstanding HER stability of the Pd‐Ni_3_N/GF in alkaline solution.

To verify the applicability of the coupling strategy, a two‐electrode coupling system was assembled for HER and **1a** electrooxidation in a large flow electrolyzer using Pd‐Ni_3_N/GF as a bifunctional electrocatalyst (Figure S23). The catholyte employed 1 m KOH, while the anolyte employed 1 m K_2_CO_3_ without or with ACT and **1a** (15 g). As illustrated in Figure 4d, the working area in the enlarged flow electrolyzer reaches 50 cm^2^ compared to the original flow electrolyzer. For comparison, a conventional water splitting system with HER and OER coupling was also established. Remarkably, the HER||**1a** oxidation system utilizing Pd‐Ni_3_N/GF electrodes requires only a cell voltage of 3.02 V to achieve a current of 5 A (Figure 4e), which is significantly lower than that of the HER||OER system (4.89 V). Figure S24 depicts a comparison of the voltages at varying currents for the large flow electrolyzer in the electrolyte without or with ACT and **1a**. The findings indicate that replacing OER with easier **1a** oxidation is an effective strategy for both energy‐saving H_2_ production and achieving value‐added products. As shown in Figure 4f, the cell voltage was maintained at 3.0–3.5 V during the constant‐current electrolysis (5 A) at a flow rate of 0.5 L/min, with an anodic potential of 1.55–1.65 V vs. RHE. The enlarged images of the electrolyte inlet and outlet show the presence of abundant H_2_ bubbles at the cathode outlet, and the **1a** oxidation occurring on the anode without O_2_ bubbles (Figure S25). As the reaction time increases, the initial sterol **1a** is observed to decrease gradually (Figure 4g), undergoing oxidation to form the target product **1b**, with minimal by‐products **1c**. In Figure 4h, for **1a** electrooxidation in the large flow electrolyzer, achieving a **1b** productivity of 47.3 mmol in 40 min (corresponding to 70.9 mmol/h) with a 99% selectivity and 76% F.E. for **1b**, demonstrates the potential of this electrocatalytic approach in large‐scale production. Meanwhile, the cathodic H_2_ productivity reached 93.1 mmol/h. Impressively, the **1a** electrooxidation space‐time yield of the Pd‐Ni_3_N/GF electrodes in a large flow electrolyzer was as high as 17.4 kg/(m^3^·h) (Figure 4i), which is significantly higher than that of the flow electrolyzer (14.8 kg/(m^3^·h)) and batch reactor (0.16 kg/(m^3^·h)). Therefore, these results demonstrate the critical role played by Pd atoms in the Pd‐Ni_3_N/GF, enhancing both HER and **1a** electrooxidation kinetics, which makes it a promising electrocatalyst for integrated energy‐saving H_2_ and value‐added steroid carbonyl products production.

### 2.4 Mechanistic Analysis of ACT‐Mediated Electrooxidation of 1a Over the Pd‐Ni_3_N/GF Electrode

**FIGURE 5:**
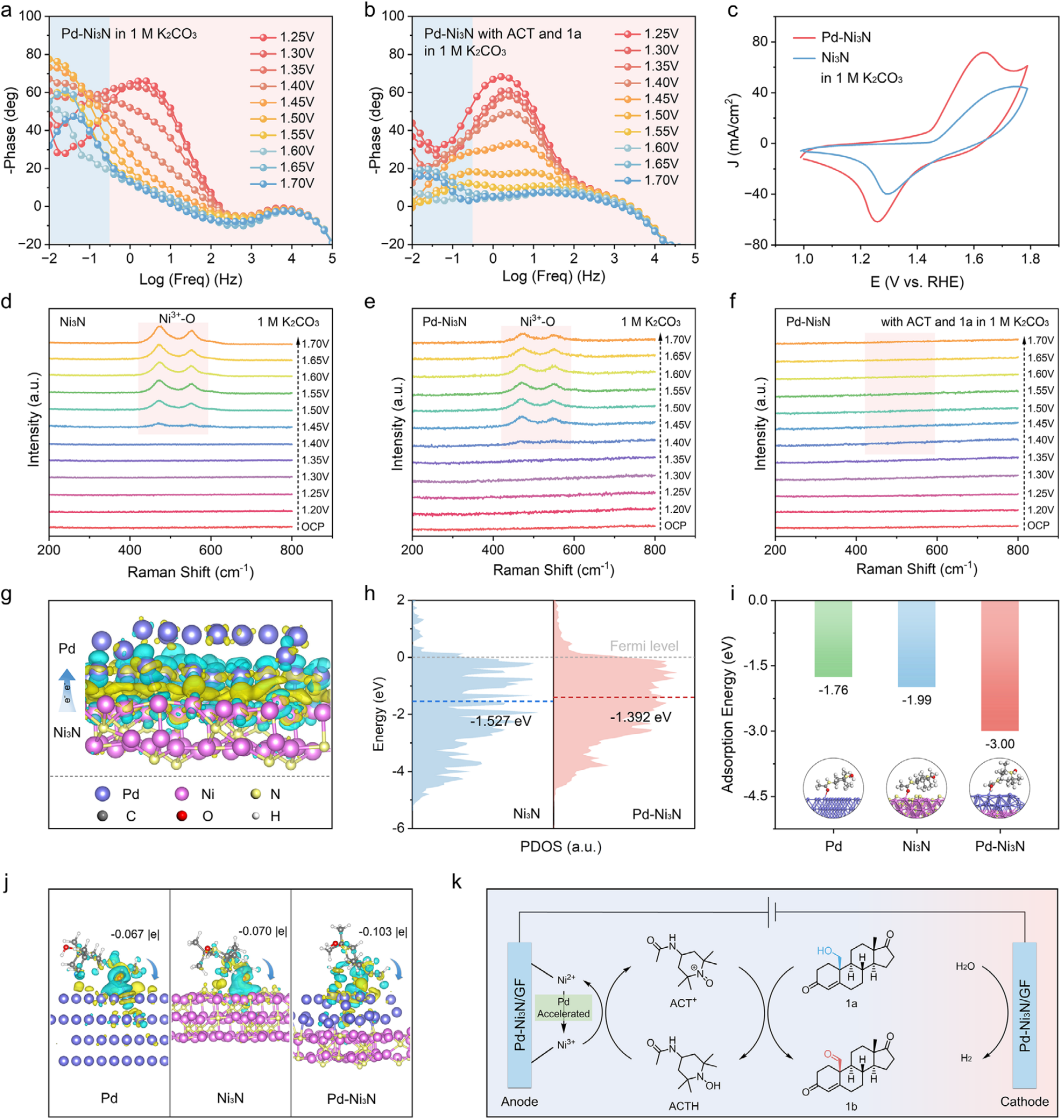
Bode plots of Pd‐Ni_3_N/GF at various voltages (increased from 1.25–1.70 V vs. RHE) in (a) 1 m K_2_CO_3_ and (b) with ACT and **1a**. (c) CV curves of Pd‐Ni_3_N/GF and Ni_3_N/GF in 1 m K_2_CO_3_. In situ Raman spectra taken on the (d) Ni_3_N and (e) Pd‐Ni_3_N surface in 1 m K_2_CO_3_ at various voltages (increased from 1.20–1.70 V vs. RHE). (f) In situ Raman spectra taken on the Pd‐Ni_3_N surface in 1 m K_2_CO_3_ and with ACT and **1a** at various voltages (increased from 1.20–1.70 V vs. RHE). (g) Charge density difference plot of Pd‐Ni_3_N. (h) Projected density of states (PDOS) diagrams of the Ni in Ni_3_N and Pd‐Ni_3_N. (i) The adsorption energy of ACTH on the surfaces of Pd, Ni_3_N, and Pd‐Ni_3_N. (j) Charge density difference of ACTH adsorption for the corresponding structures of Pd, Ni_3_N, and Pd‐Ni_3_N. (k) Proposed mechanism for the ACT‐mediated electrooxidation of **1a** and H_2_ production over the Pd‐Ni_3_N/GF electrode.

To elucidate the in‐depth mechanism insight toward the ACT‐mediated electrooxidation of **1a** over Pd‐Ni_3_N/GF, a series of in situ characterization techniques was systematically performed. In situ EIS was employed to explore the catalytic kinetics, electron transfer, and the electrochemical interface behavior at different potentials [55]. The high frequency region in Bode plots corresponds to electrode interior oxidation, whereas the low frequency region reflects non‐homogeneous charge distribution, namely the generation of oxidation species at the electrode interface [56]. In the absence of **1a** and ACT, the interface phase angle of the corresponding to the Bode plot decreases with increasing potential for the Pd‐Ni_3_N/GF (Figure 5a), implying an enhanced reaction rate. With a further increase in the potential to 1.70 V vs. RHE, a distinct transition peak was observed in the phase angle, signifying the occurrence of the oxygen evolution, which aligns with its OER onset potential. The incorporation of ACT into the electrolyte led to a decline in phase angle at all potentials and a shift of the phase angle toward the high‐frequency region compared to the electrolyte alone (Figure S26), which can be attributed to the accelerated charge transfer of the interfacial reaction. As depicted in Figure 5b, the phase angle peak decreases and shifts to a higher frequency as the potential increases after adding **1a** and ACT, implying that more absorbed **1a** molecules were rapidly oxidized, resulting in accelerated interfacial charge transfer. The corresponding Nyquist plots revealed that the presence of ACT and **1a** in the electrolyte resulted in the formation of smaller semicircles compared to the blank electrolyte (Figure S27), further confirming the reduced charge transfer resistance, thereby facilitating the ACT‐mediated electrooxidation of **1a** on the Pd‐Ni_3_N/GF electrode.

Moreover, CV tests and UV–vis spectral experiments were performed to further investigate the interaction between Pd‐Ni_3_N/GF and ACT [16]. As illustrated in Figure S28a, using GF as the working electrode in 1 m K_2_CO_3_ electrolyte, the isolated ACT exhibited a pair of redox peaks at 1.60 and 1.45 V vs. RHE, corresponding to the transitions between ACT/ACT^+^ and ACT^+^/ACT, respectively. The Pd‐Ni_3_N/GF also demonstrated redox peaks attributable to the Ni^2+^/Ni^3+^ couple in 1 m K_2_CO_3_ solution. Upon introducing the Pd‐Ni_3_N/GF electrode into the ACT‐containing electrolyte, the peak signals of ACT/ACT^+^ significantly increased. Subsequently, the above Pd‐Ni_3_N/GF electrode was washed multiple times with MeCN and H_2_O to remove ACT, followed by repeated CV tests. The treated electrode exhibited higher current densities than the pristine Pd‐Ni_3_N/GF, indicating persistent adsorption of ACT on the Pd‐Ni_3_N/GF surface. To exclude potential interference from the GF substrate on ACT adsorption, control CV experiments were conducted under identical conditions. As depicted in Figure S28b, the current densities of ACT‐immersed GF electrodes remained comparable to those of the pristine GF, demonstrating negligible ACT adsorption on the GF substrate and highlighting the critical role of Pd‐Ni_3_N/GF in facilitating ACT adsorption. Besides, UV–vis spectroscopic analysis shows that Pd‐Ni_3_N/GF (Ni^2+^) exhibited negligible variation in solution absorbance after immersion in ACT solution (Figure S29a). Similarly, no UV–vis absorption signal for ACT was observed with the GF substrate alone, consistent with the CV results. In contrast, a significant decrease in absorbance occurred when the Pd‐Ni_3_N/GF (Ni^3+^) catalyst was immersed in ACT solution, suggesting potential adsorption or consumption of ACT by the Pd‐Ni_3_N/GF (Ni^3+^) electrode. Obviously, the adsorption of Pd‐Ni_3_N/GF (Ni^3+^) toward the ACT solution was stronger than that of Ni_3_N/GF (Ni^3+^), further demonstrating that the introduction of Pd promotes the generation of Ni^3+^ species (Figure S29b). This phenomenon is also evident in the CV curve, the introducing Pd not only induces oxidation of Ni^2+^‐OH in Ni_3_N at lower potentials but also significantly accelerates the rate of formation of Ni^3+^‐O in Pd‐Ni_3_N, as evidenced by the elevated peak current density (Figure 5c).

Additionally, in situ Raman spectra were further applied to monitor the surface structure evolution of the Ni_3_N and Pd‐Ni_3_N during the electrooxidation processes at different potentials. Upon increasing the potential, two emerging Raman peaks at 474 and 552 cm^−1^ were observed at 1.45 V vs. RHE in Ni_3_N/GF electrocatalyst (Figure 5d), belonging to the δ(Ni^3+^‐O) and ν(Ni^3+^‐O) vibrations of NiOOH species, such surface reconstruction phenomena have been evidenced in numerous other Ni‐based catalyst systems [8, 57, 58]. The lack of Ni^2+^‐O vibrational peaks in the Raman spectra resulted from the limited sensitivity of the spectrometer and the inertness of the Ni^2+^‐O vibrational modes. Notably, these Raman peak signals of Ni^3+^‐O were detected when the potential reached 1.40 V vs. RHE for Pd‐Ni_3_N/GF (Figure 5e), demonstrating that the active NiOOH species formed more readily at lower potentials in the Pd‐Ni_3_N/GF electrocatalyst compared to Ni_3_N/GF, which is consistent with the CV results (Figure 5c). This suggests that the introduction of Pd facilitates Ni^3+^‐O formation during oxidative reactions. Upon introducing ACT into the electrolyte, the Ni^3+^‐O signals remained undetectable until the applied potential exceeded 1.50 V vs. RHE (Figure S30), implying rapid consumption of electrogenerated NiOOH by ACT. At potentials surpassing 1.50 V vs. RHE, limited ACT adsorption capacity prevented complete NiOOH consumption, leading to the reappearance of NiOOH species. The Ni^3+^‐O peak signal completely disappears even at an applied potential of 1.70 V vs. RHE after adding both ACT and **1a** in the K_2_CO_3_ electrolyte (Figure 5f).

Additionally, the density functional theory (DFT) calculations were performed to further gain mechanistic insights into the role of introducing Pd in Ni_3_N. The optimized model structures of Pd‐Ni_3_N, Ni_3_N, and Pd were constructed based on the experimental results (Figure S31). The electronic changes at the Pd‐Ni_3_N interface were indicated by the conducting charge density difference (CDD), where the blue color represents electron deficiency, and the yellow color indicates electron accumulation (Figure 5g). The charge density of Ni_3_N was weakened after introducing Pd, which stabilizes Ni species in a higher oxidation state. It was clearly found that the strong interaction between Pd and Ni_3_N, with electron donation from Ni_3_N to the Pd side, leads to electron accumulation at the interface on the Pd side, further resulting in a reduction in the charge density of Ni_3_N and an increase in the oxidation state of elemental nickel, which aligns well with the experimental findings. The projected density of states (PDOS) diagrams of the element Ni in Ni_3_N and Pd‐Ni_3_N were plotted as shown in Figure 5h. The closer shift of the d‐band center of Ni toward the Fermi level in Pd‐Ni_3_N (−1.392 eV) compared to Ni_3_N (−1.527 eV). Interestingly, ACTH molecules underwent chemical adsorption through keto‐oxygen interactions with Pd‐Ni_3_N on the surface. The Pd‐Ni_3_N shows a lower adsorption energy (−3.00 eV) in ACTH compared to Ni_3_N (−1.99 eV, Figure 5i; Figure S32), indicating that Pd‐Ni_3_N has a strong ACTH adsorption capacity, which facilitates the initial adsorption and activation of ACTH. The CDD calculation also reveals that ACTH adsorbed on Pd‐Ni_3_N transfers more charge (−0.103|e|) to the Pd side than on Pd (111) (−0.067|e|) and Ni_3_N (111) (−0.070|e|) surfaces, which accelerates the oxidation of ACTH to ACT^+^ (Figure 5j). Integrated results of CV, UV–vis spectroscopy, in situ Raman, constant current electrolysis, and DFT calculation, a proposed reaction mechanism for the ACT‐mediated electrooxidation of **1a** on the Pd‐Ni_3_N/GF anode is presented [16, 40], whereby Pd in Pd‐Ni_3_N/GF promotes the formation of NiOOH, which accelerates the adsorption and oxidation of ACT or ACTH to ACT^+^, and finally ACT^+^ serves for the rapid oxidation of **1a**–**1b** (Figure 5h).

## 3 Conclusions

In summary, we demonstrated a versatile and efficient strategy for the selective electrooxidation of sterol **1a** to value‐added **1b** and H_2_ production at high current densities by using a Pd‐Ni_3_N/GF electrocatalyst. At anode, the integrating Pd‐Ni_3_N and ACT exhibited excellent **1a** electrooxidation activity with a high selectivity (99%), yield (98%), and F.E. (88%). Additionally, the Pd‐Ni_3_N/GF exhibits superior HER activity, with a low overpotential of 18 mV (−10 mA/cm^2^) and a Tafel slope of 66 mV/dec. Subsequently, we designed a large flow electrolyzer using Pd‐Ni_3_N/GF for the co‐production of **1b**‐H_2_, which requires a low voltage of 3.02 V to achieve an industrially relevant current of 5 A, attaining productivities of 70.9 and 93.1 mmol/h for **1b** and H_2_, respectively. The space‐time yield of the **1a** electrooxidation in a large flow electrolyzer was 17.4 kg/(m^3^·h), outperforming both the flow electrolyzer and batch reactor, highlighting its potential for practical applications. Additionally, the experimental and theoretical analyses demonstrated that the Pd acts as an “electron pump” that accelerates the formation of high‐valent Ni^3+^ active centers, facilitating adsorption and activation of ACT or ACTH, thereby boosting the catalytic activity of sterol oxidation. This work offers a novel strategy for the design and synthesis of highly efficient electrocatalysts and electrolyzers toward the electrosynthesis of valuable steroid intermediates coupled with H_2_ production.

## Conflicts of Interest

The authors declare no conflicts of interest.

## Supporting information




**Supporting File**: advs74527‐sup‐0001‐SuppMat.docx

## Data Availability

The data that support the findings of this study are available in the supplementary material of this article.
