# Supplementary material for: Rational Design of Ni3+‐Rich Nickel Nitride Enables Paired Electrooxidation of Sterol and Hydrogen Evolution in a Flow Electrolyzer
Source: Adv Sci (Weinh). 2026 Feb 19;13(25):e74527. doi: 10.1002/advs.74527 (PMC13137849; doi:10.1002/advs.74527)
Supplement: Supplementary file 1 — Supporting File: advs74527‐sup‐0001‐SuppMat.docx [file ADVS-13-e74527-s001.docx]

Copyright WILEY-VCH Verlag GmbH & Co. KGaA, 69469 Weinheim, Germany, 2013.

Supporting Information

**Rational Design of Ni^3+^-Rich Nickel Nitride Enables Paired Electrooxidation of Sterol and Hydrogen Evolution in a Flow Electrolyzer**

*Suiqin Li^a,#^, Jieyu Wang^b,#^, Ge Feng^c,#^, Jiahui He^b^, Kai Li^b^, Lihao Liu^b^, Yuhang Wang^b^, Zixian Jia^a^, Yanfei Xu^a^, Wenwu Zhong^a,*^, Xing Zhong^b,*^, Jianguo Wang^b,*^*

^a^Zhejiang Key Laboratory of Functional ionic membrane Materials and Technology for Hydrogen Production, Shaoxing University, Shaoxing 312000, China.

^b^State Key Laboratory of Green Chemical Synthesis and Conversion, Zhejiang Key Laboratory of Surface and Interface Science and Engineering for Catalysts, College of Chemical Engineering, Zhejiang University of Technology, Hangzhou 310032, China.

^c^School of Environment and Natural Resources, Zhejiang University of Science & Technology, Hangzhou 310023, China.

^#^S. Li, J. Wang and G. Feng contribute to this work equally.

*Correspondence and requests for materials should be addressed to W.W.Z. (zhongww@tzc.edu.cn), X.Z. (email: zhongx@zjut.edu.cn), or J.G.W. (email: jgw@zjut.edu.cn).

**1. Experimental Section**

**1.1. Synthesis of Ni(OH)F/GF**

The graphite felt (GF, dimensions: 3 cm × 3.5 cm × 0.6 cm) was pretreated via calcination in a laboratory muffle furnace. The GF sample was heated from room temperature to 500°C under ambient air atmosphere and maintained at this temperature for 30 min. Subsequently, the Ni(OH)F/GF precursor was synthesized via a hydrothermal method. In brief, stoichiometric amounts of Ni(NO_3_)_2_·6H_2_O (2 mmol), NH_4_F (8 mmol) and urea (12 mmol) were dissolved in 80 mL of deionized water under continuous stirring. The above solution and GF were transferred into a 100 mL Teflon-lined autoclave and subjected to hydrothermal reaction under the 120°C for 12 h. After cooling to room temperature, the Ni(OH)F/GF precursor was thoroughly washed with deionized water and ethanol to remove residual impurities, and vacuum-dried overnight at 70°C.

**1.2. Synthesis of Ni_3_N/GF**

For the preparation of Ni_3_N/GF, the Ni(OH)F/GF precursor and 800 mg of urea powder were loaded into a crucible within a tubular furnace. The urea was positioned upstream of the precursor to facilitate controlled ammonia release during thermal decomposition. Under a continuous Ar flow, the sample was annealed at 450°C for 3 h with a heating rate of 5°C/min, followed by natural cooling to room temperature to obtain the final Ni_3_N/GF product.

**1.3. Synthesis of Pd-Ni_3_N/GF**

The Pd-Ni_3_N/GF was prepared by a pyrolysis method. The prepared Ni_3_N/GF composite and 120 mg of palladium acetylacetonate (Pd(acac)_2_) powder (corresponding to a Pd loading of 5%) were placed together in a crucible inside a tube furnace, with the Pd(acac)_2_ positioned upstream of the Ni_3_N/GF. The sample was heated to 350 °C under a flowing argon (Ar) atmosphere at a controlled ramp rate of 5 °C/min and then annealed at this temperature for 3 h in order to synthesise Pd-Ni_3_N/GF. The mass loading of the Pd-Ni_3_N/GF catalyst was determined to be in the range of 1-2 mg/cm^2^ by measuring the weight difference between the pristine GF and the GF coated with Pd-Ni_3_N. The precise loading amount of Pd was obtained by inductively coupled plasma mass spectrometry (ICP-MS). For comparison, Pd-Ni_3_N/GF catalysts with Pd loadings of 2.5%, 7.5%, and 10% were also prepared using the same procedure. Additionally, Pd nanoparticles were directly supported on GF using the same pyrolysis method as in the aforementioned experiment to form Pd/GF as a comparison catalyst, with the same Pd loading as in Pd-Ni_3_N/GF (5% Pd loading).

**2. Material Characterizations**

The morphology characteristics of the samples were investigated using scanning electron microscopy (SEM, Hitachi FE-SEM S-4700) and transmission electron microscopy (TEM, JEM-ARM300F). X-ray diffraction (XRD) with graphite-monochromatized Cu Kα radiation (λ = 1.54 Å) was employed to analyze the crystal structure of the samples. X-ray photoelectron spectroscopy (XPS, Thermo Scientific ESCALAB 250Xi) was utilized to determine the chemical composition and bonding states of the samples. The elemental composition of the sample was quantified via inductively coupled plasma mass spectrometry (ICP-MS, PerkinElmer). In situ Raman spectroscopy was performed using a confocal Raman imaging microscope (Renishaw InVia) equipped with a 532 nm laser. Measurements were conducted in a three-electrode system controlled by an Ivium-n-Stat electrochemical workstation, with potentials ranging from 1.20 to 1.70 V vs. RHE.

**3. Electrochemical measurements**

All electrochemical measurements, Cyclic voltammetry (CV), linear sweep voltammetry (LSV), and chronopotentiometry (CP), were conducted using an Ivium-n-Stat electrochemical workstation. The Pd-Ni_3_N/GF (1 cm × 1 cm) was directly employed as the working electrode, while a Pt wire (1.5 cm × 1.5 cm) and a standard Hg/HgO electrode served as the counter electrode and reference electrode, respectively. Oxygen evolution reaction (OER) was performed in a 1 M K_2_CO_3_ electrolyte solution, and the hydrogen evolution reaction (HER) was performed in a KOH electrolyte solution, with a carbon rod serving as the counter electrode in the HER. The electrooxidation of sterol was measured in a mixed solution of 1 M K_2_CO_3_ with MeCN containing **1a** (1.5 g, 5 mmol) and ACT (10 mol%), the volume ratio of 1 M K_2_CO_3_ to MeCN in the mixed solution was 7:3. A bare graphite felt (GF) served as the working electrode for measuring the LSV curves and for comparing the yield of ACT, while maintaining dimensions comparable to those of the Pd-Ni_3_N/GF electrode (1 cm × 1 cm). The scan rate for CV was kept at 50 mV/s, and the LSV was kept at 10 mV/s in a batch reactor and flow electrolyzer, with 80% iR compensation for HER. All measured potentials were converted to the reversible hydrogen electrode (RHE) using the Nernst equation: E_(RHE)_ = E_(Hg/HgO)_ + 0.0592 pH + 0.098 V. The electrochemical surface area (ECSA) was evaluated via CV by measuring the double-layer capacitance (C_dl_) within a non-Faradaic potential window, utilizing scan rates ranging from 20 to 120 mV/s. Electrochemical impedance spectroscopy (EIS) was carried out over a frequency range of 100,000 to 0.1 Hz, with applied potentials from 1.25 to 1.70 V vs. RHE in 0.05 V increments.

The constant-current electrolysis experiment was conducted in a flow electrolyzer, with an electrode area of approximately 10 cm^2^, where a Nafion 417 membrane (Suzhou Thinkre New Material Co., Ltd.) separated the anode from the cathode, with the anodic potential monitored using a standard Hg/HgO reference electrode. The anolyte consisted of a mixed solution (100 mL) of 1 M K_2_CO_3_ and acetonitrile (MeCN) in a 7:3 volume ratio, containing sterol **1a** (1.5 g, 5 mmol) and ACT (10 mol%), while 100 mL of 1 M KOH served as the catholyte. A magnetic pump and peristaltic pump were employed to ensure uniform mixing of the reaction solution and continuous electrolyte circulation within the reaction system, respectively. The electrolysis was performed at a constant current of 1 A, corresponding to a current density of 100 mA/cm^2^. The final product was characterized and quantified by high-performance liquid chromatography (HPLC).

The scaled-up electrolysis experiment was performed in a large flow electrolyzer with a Nafion 417 membrane separated the anode from the cathode, where the working electrode surface area of approximately 50 cm^2^. A standard Hg/HgO reference electrode was employed to monitor the anode potential. The anolyte consisted of 1 L of 1 M K_2_CO_3_ and MeCN mixed solution (7:3 ratio) containing sterol **1a** (15 g, 50 mmol) and ACT (10 mol%), while the catholyte comprised 1 M KOH solution (1 L). To ensure enhanced homogeneity of the reaction mixture, a mechanical stirrer was implemented for continuous agitation during the electrochemical process. To ensure homogeneous mixing of the solution, a mechanical stirrer was implemented throughout the process. The electrolysis reaction was performed under a constant current of 5 A, corresponding to a current density of 100 mA/cm^2^.

The comparative electrolysis experiment was conducted in a batch reactor using a three-electrode system, where the Pd-Ni_3_N/GF (10 cm^2^) was directly employed as the working electrode, with a Pt sheet and Hg/HgO serving as the counter electrode and reference electrode, respectively. The electrolyte solution comprised a mixture of 100 mL 1 M K_2_CO_3_ and MeCN (7:3 ratio), containing sterol **1a** (1.5 g, 5 mmol) and ACT (10 mol%). Magnetic stirring was implemented to ensure homogeneous distribution of the electrolyte mixture during the electrochemical process. The electrolysis was performed under a constant current of 100 mA, corresponding to an current density of 10 mA/cm^2^.

The yield of oxidation products was calculated using equations (1):

$$\mathrm{Yield}\left( \% \right)= \frac{mol of main product formed}{mol of initial substrate} \times100\% , (1)$$

The Faradaic efficiency (F.E.) formation was calculated using equations (2):

$$F.E. \left( \% \right)= \frac{mol of main product formed}{total charge passed / (n \times F)} \times100\% , (2)$$

Here, *n* is number of electron transfers, *F* is the Faraday constant (96485 C/mol).

The space-time yield (Y_ST_, kg/(m^3^·h)) for electrocatalytic oxidation of sterols was calculated as shown in (3)^[1]^:

$$Y_{ST} \left( kg/\left( m^{3}\cdot h \right) \right) = \frac{m}{t\times V_{R}} , (3)$$

Here, *m* is the quality of products (kg), *t* is the reaction time (h), *V_R_* is the volume of reactor (m^3^).

**4. Theoretical calculations**

Density functional theory (DFT) calculations were carried out with the Perdew-Burke-Ernzerhof (PBE)^[2]^ functional in the Vienna ab initio simulation package (VASP).^[3]^ The projector augmented-wave (PAW) method^[4]^ was employed with a plane-wave basis set cutoff of 450 eV. The convergence criterion of 10^-5^ eV was used for electronic self-consistency, while ionic relaxation calculation was converged until 0.05 eV/Å. The Pd(111) and Ni_3_N(111) surfaces were modeled using a slab geometry with a periodically repeated (2 × 2) unit cell. The constructs of Pd-Ni_3_N were based on the Ni_3_N(111). To avoid interactions between adjacent slabs, the vacuum layer of 15 Å was placed along the z-direction for all structures. The gamma-centered k-points mesh was set up with 2×2×1 for all structures.

The ACTH adsorption energy (E_ACTH-adzs_) can be calculated as follows:

E_ACTH-ads_ = E_total_ - (E_slab_ + E_ACTH_)

Here, E_total_ is the energy of the optimized slab with the adsorbate, E_slab_ is the energy of the Pd, Ni_3_N, and Pd-Ni_3_N modelling, and E_ACTH_ is the energy of the ACTH specie.

**5. Supplementary Results**


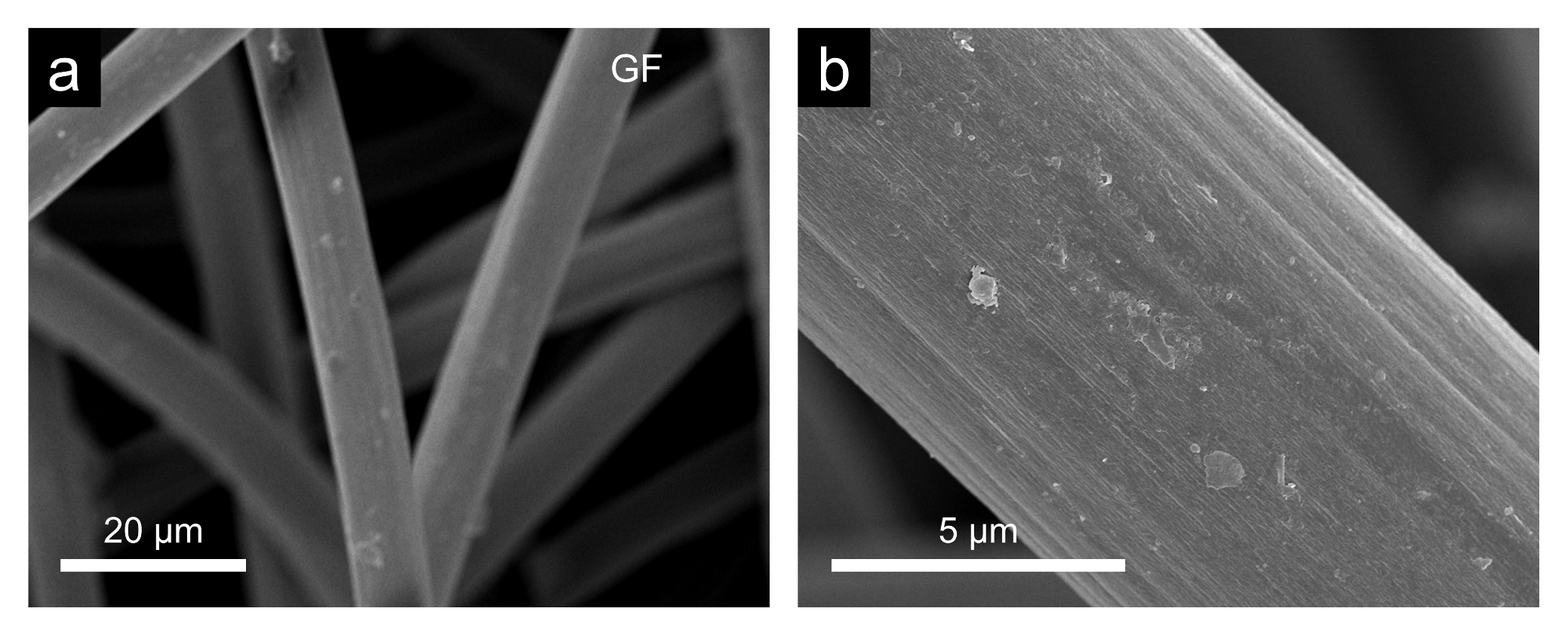


**Figure S1.** SEM of graphite felt (GF).


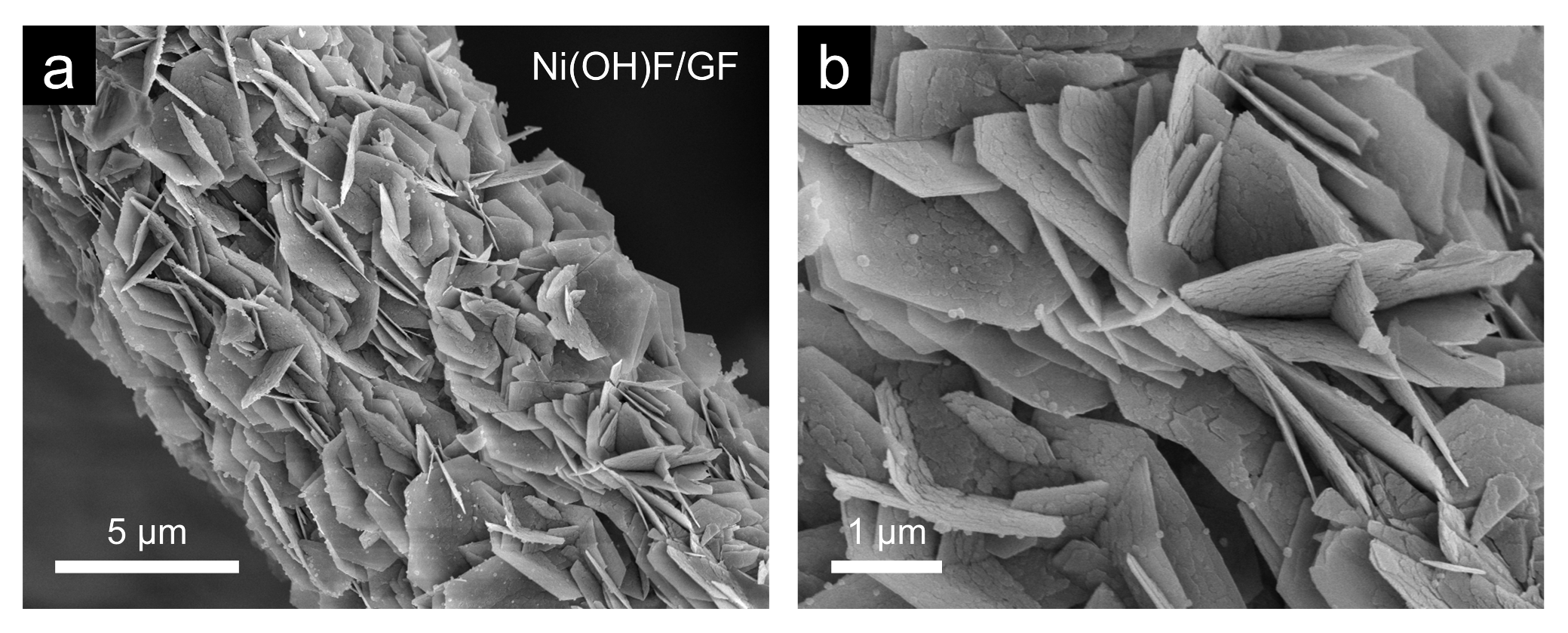


**Figure S2.** SEM of Ni(OH)F/GF.


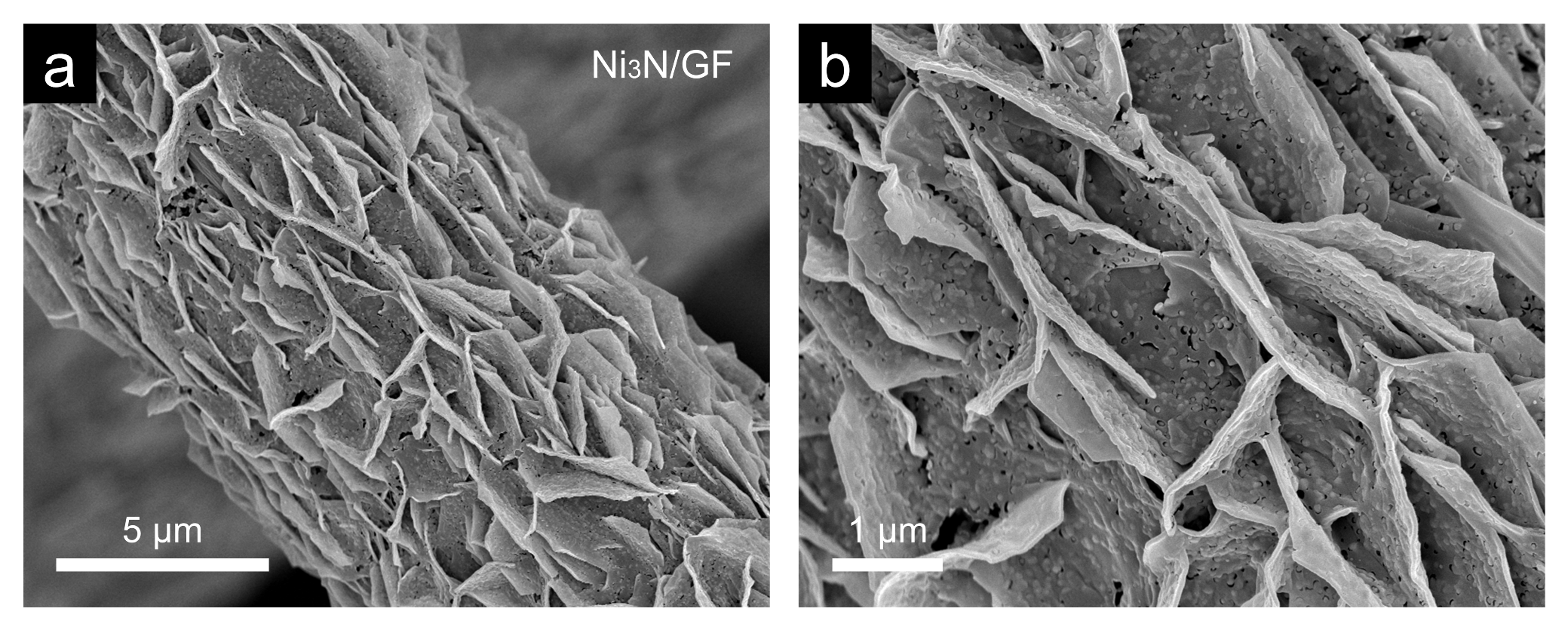


**Figure S3.** SEM of Ni_3_N/GF.


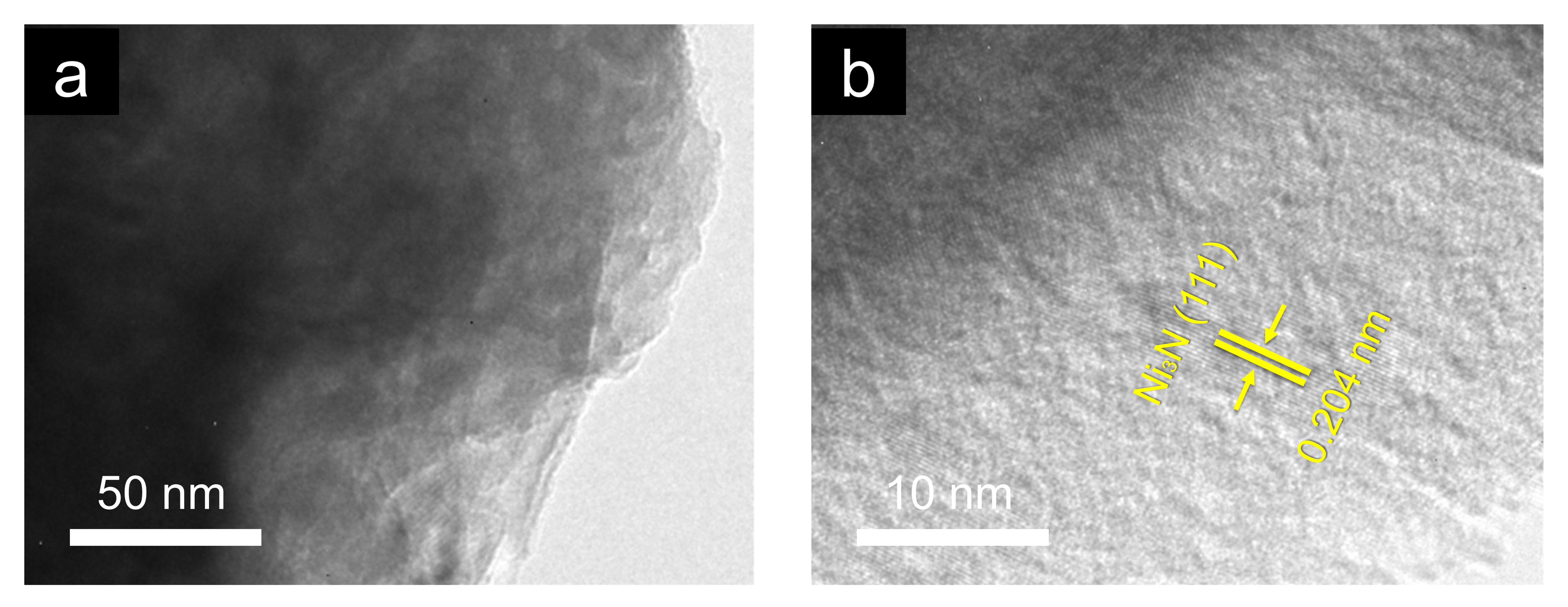


**Figure S4.** TEM of Ni_3_N/GF.


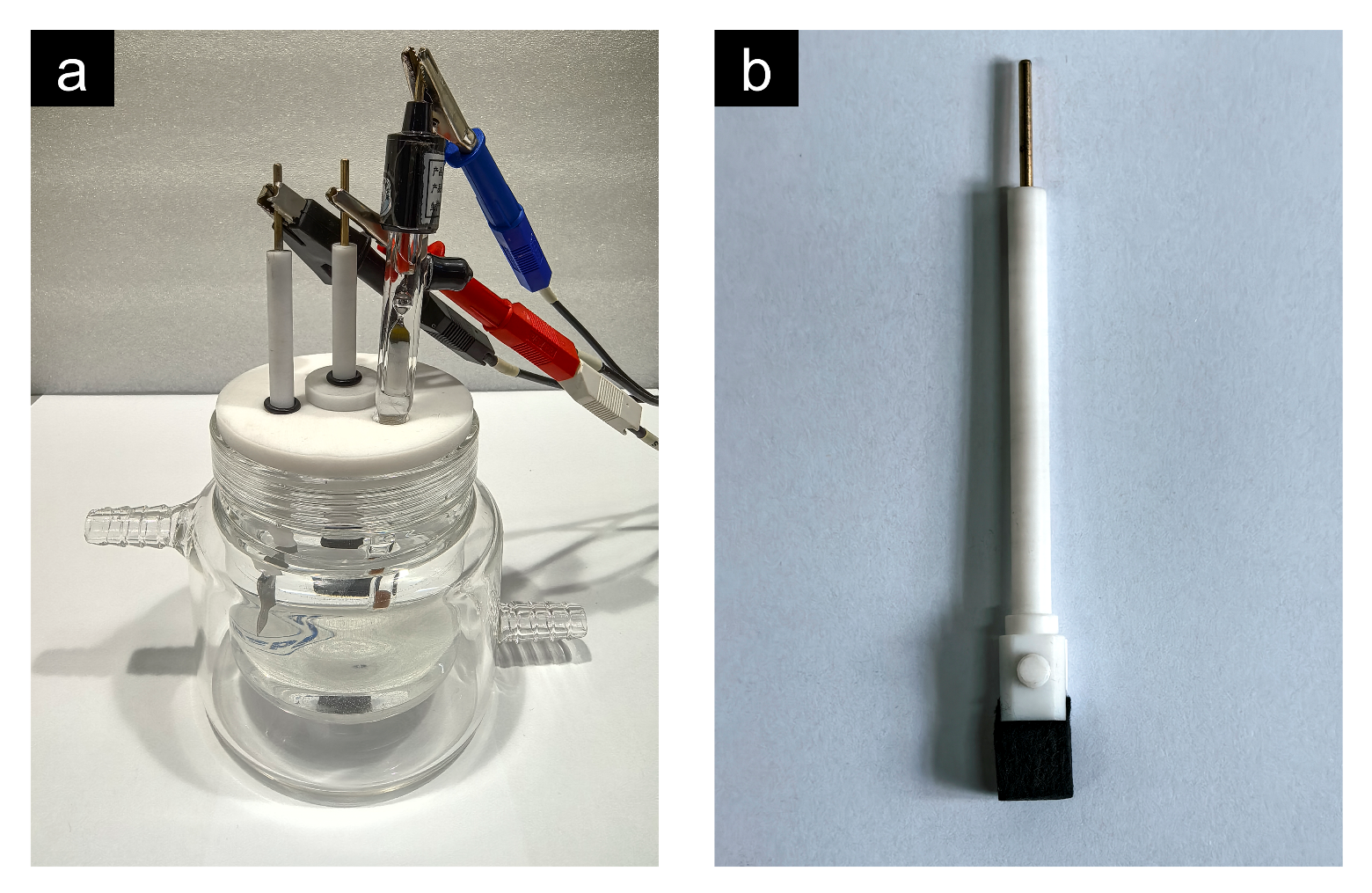


**Figure S5.** (a) A standard three-electrode system in 1 M K_2_CO_3_ electrolyte in batch reactor. (b) Working electrode of Pd-Ni_3_N/GF.


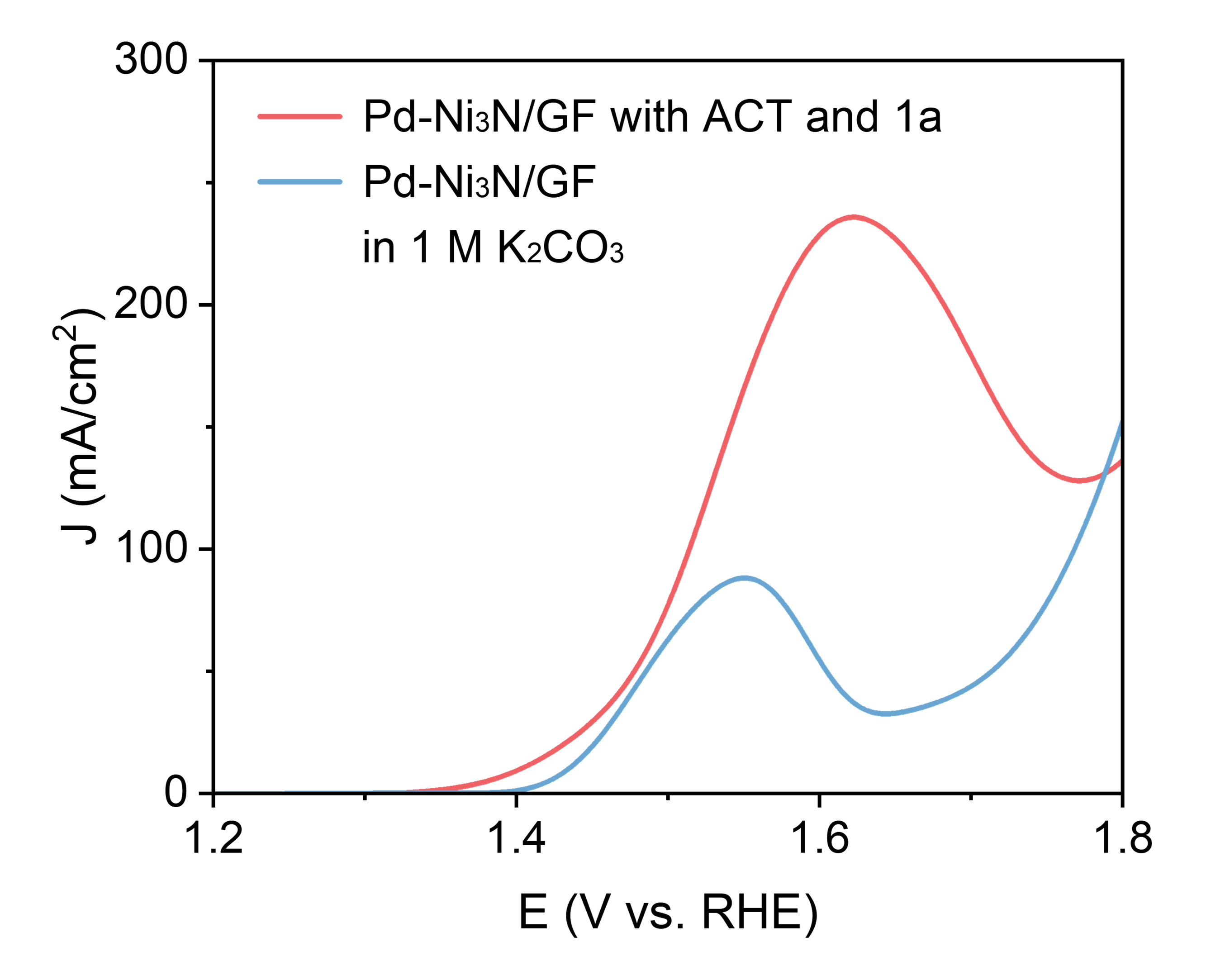


**Figure S6.** LSV curves of Pd-Ni_3_N/GF in 1 M K_2_CO_3_ electrolyte without and with ACT + **1a** in batch reactor.


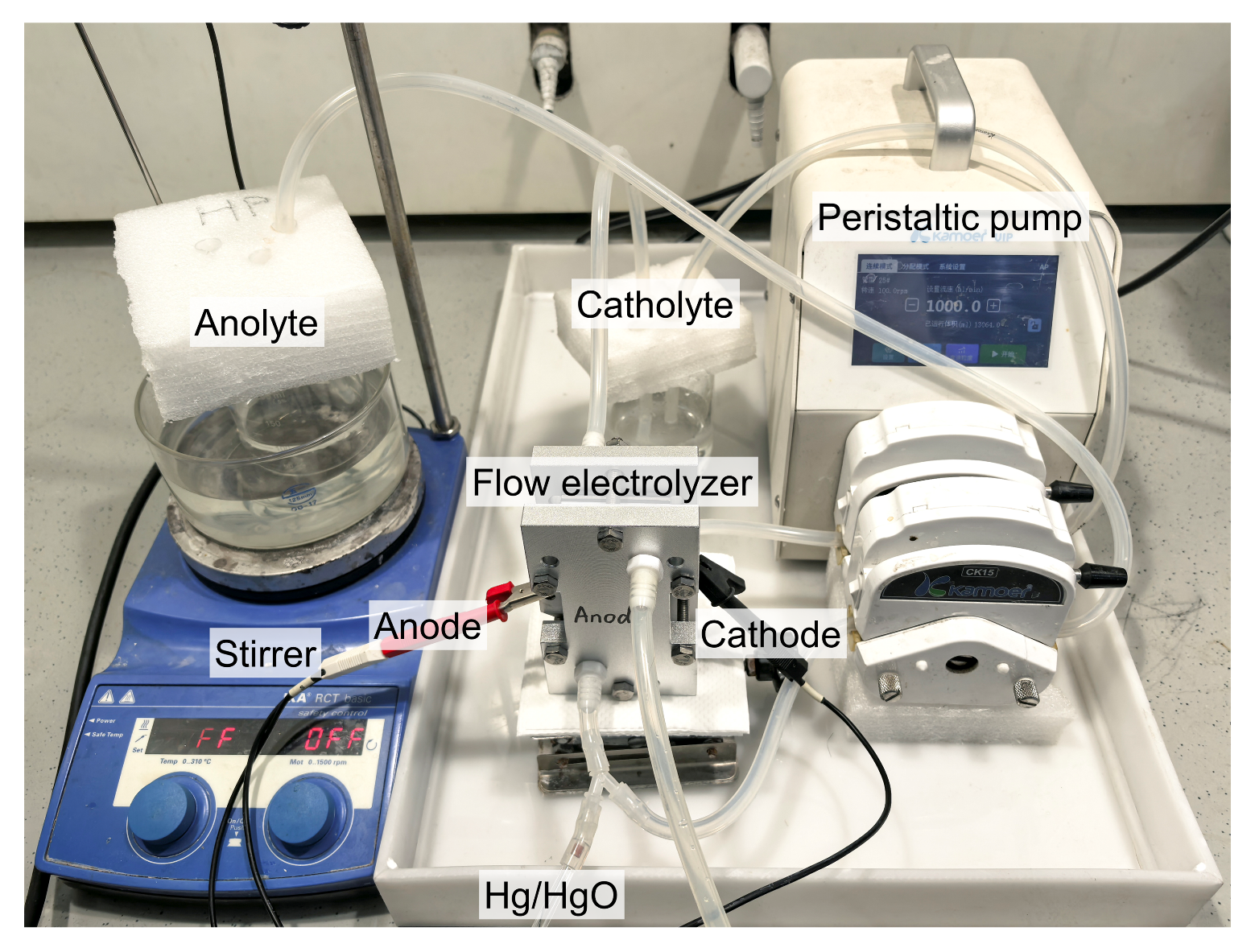


**Figure S7.** Electrolysis setup of flow electrolyzer for **1a** oxidation.


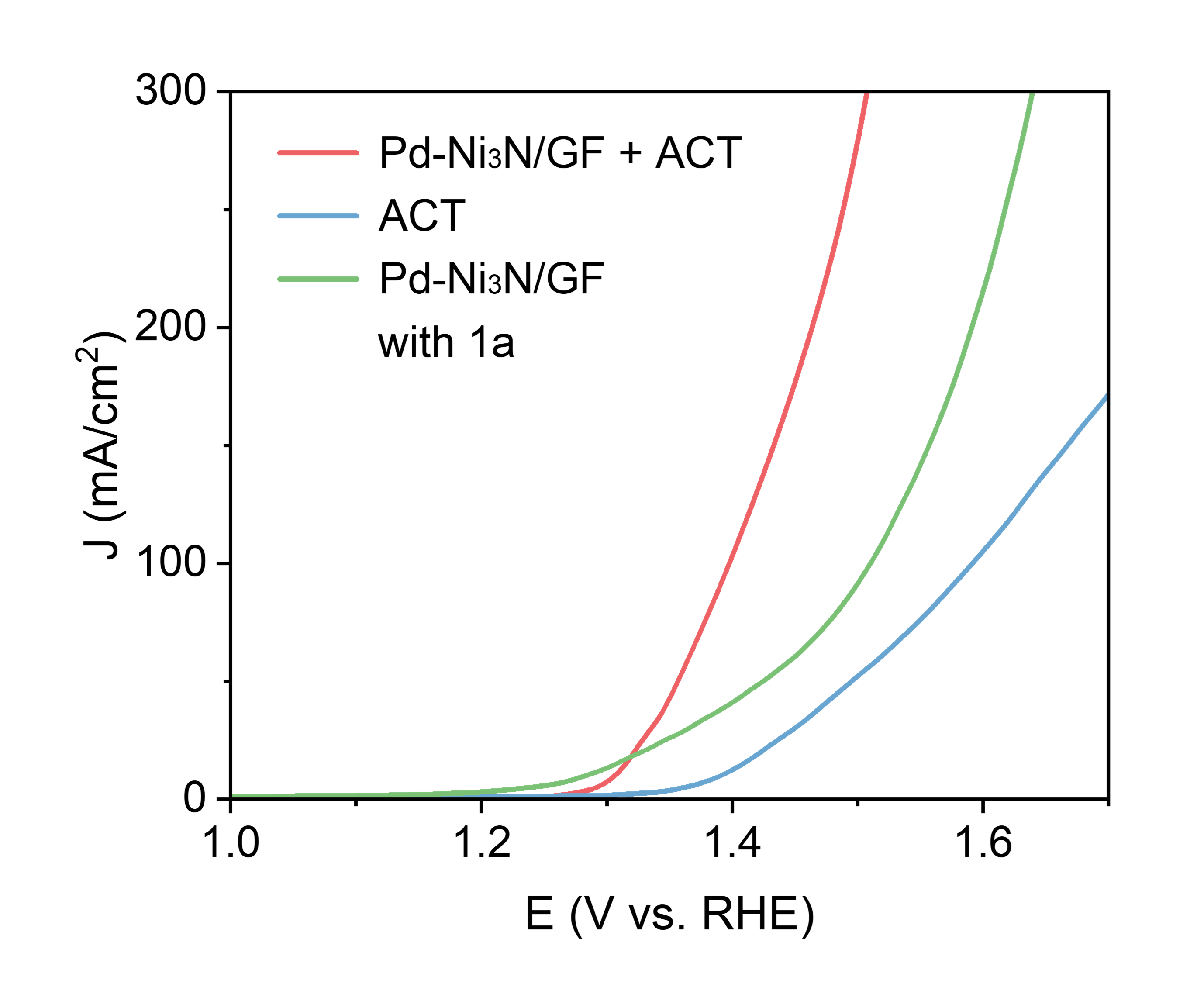


**Figure S8.** LSV curves of Pd-Ni_3_N/GF, ACT, and Pd-Ni_3_N/GF + ACT in 1 M K_2_CO_3_ electrolyte in the presence of **1a** in a flow electrolyzer. A bare GF served as the working electrode to record the LSV curves of ACT. Scan rate 10 mV/s.


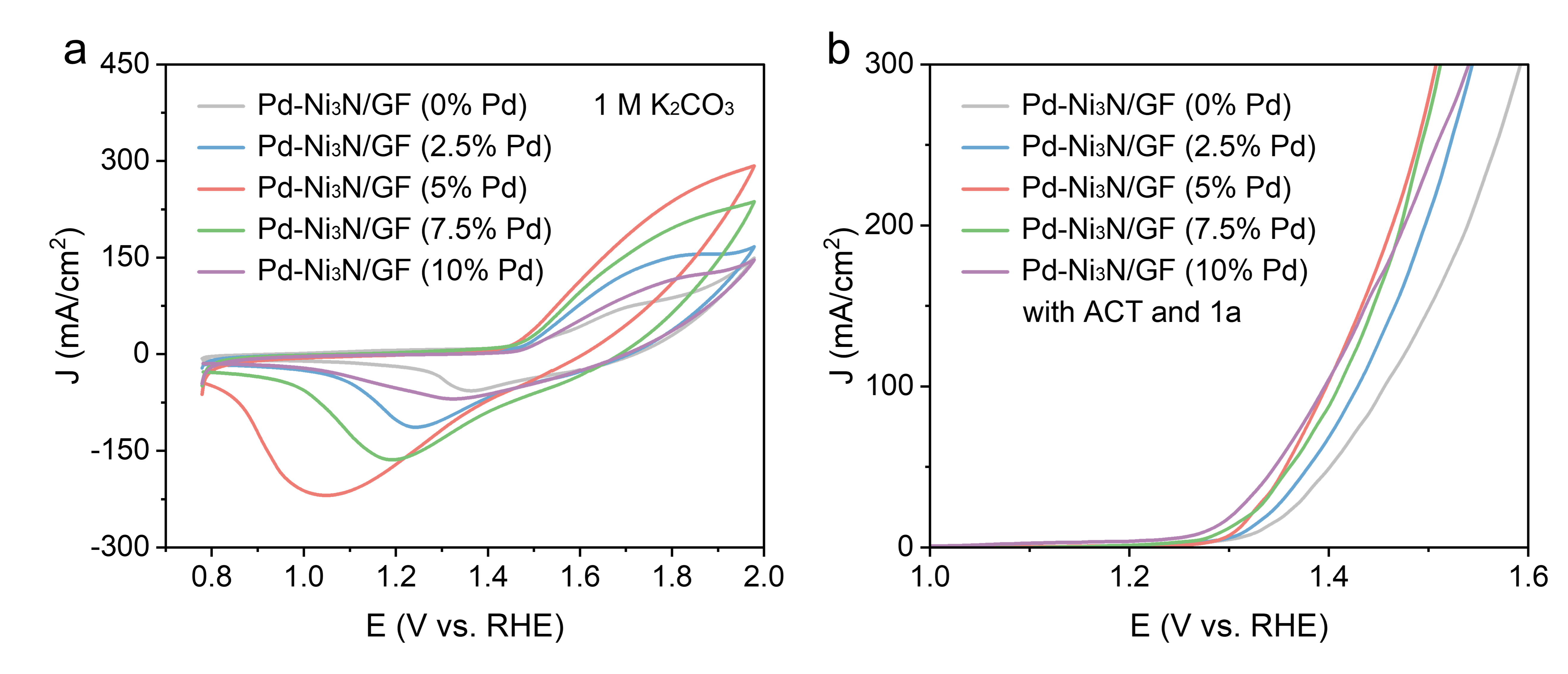


**Figure S9.** (a) CV curves of Pd-Ni_3_N/GF with different Pd mass loading in 1 M K_2_CO_3_ electrolyte in batch reactor. Scan rate 50 mV/s. (b) LSV curves of Pd-Ni_3_N/GF with different Pd mass loading in 1 M K_2_CO_3_ electrolyte in the presence of **1a** and ACT in flow electrolyzer. Scan rate 10 mV/s.


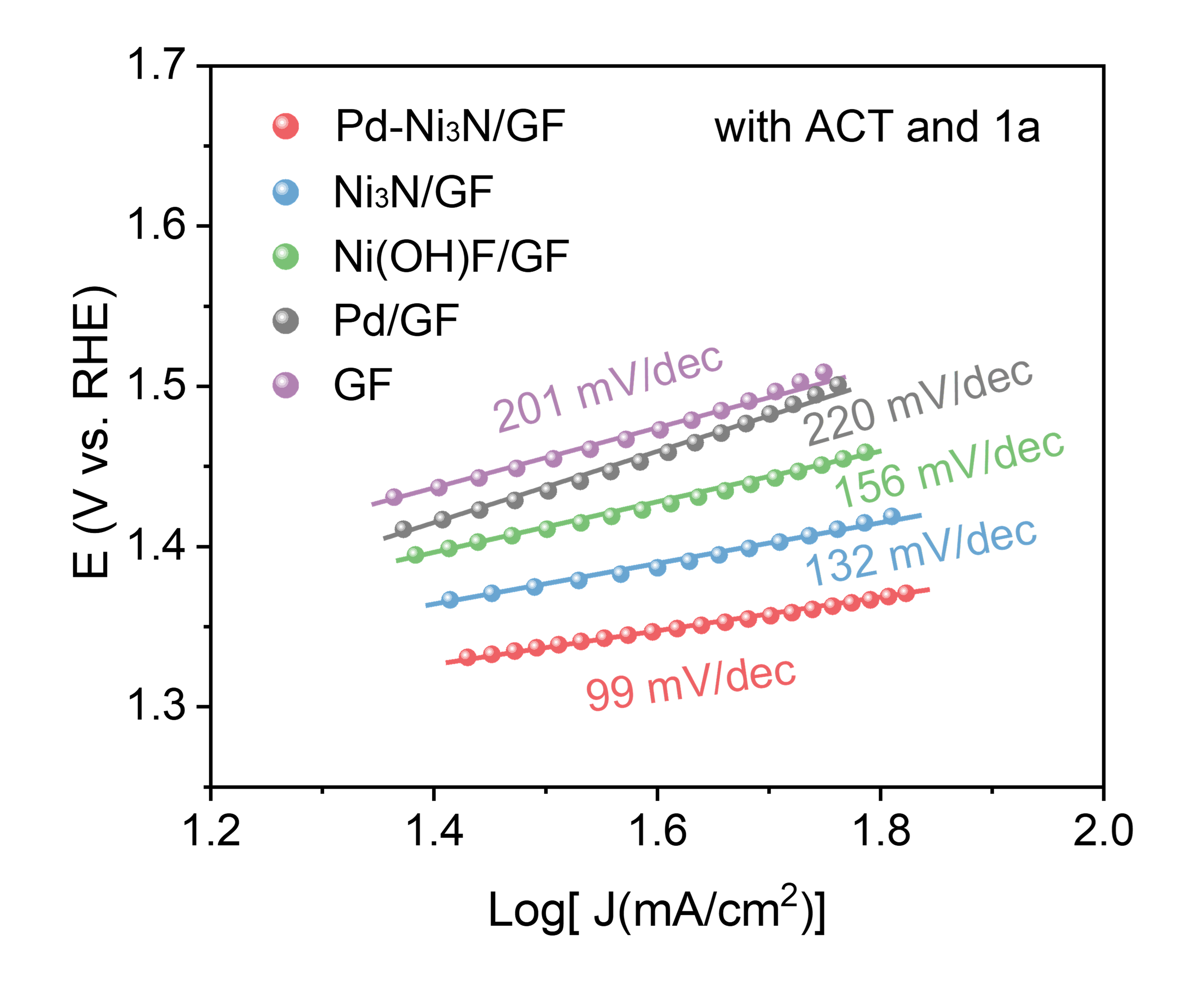


**Figure S10.** The Tafel plots of Pd-Ni_3_N/GF, Ni_3_N/GF, Ni(OH)F/GF, Pd/GF, and GF in 1 M K_2_CO_3_ in the presence of **1a** and ACT in flow electrolyzer.


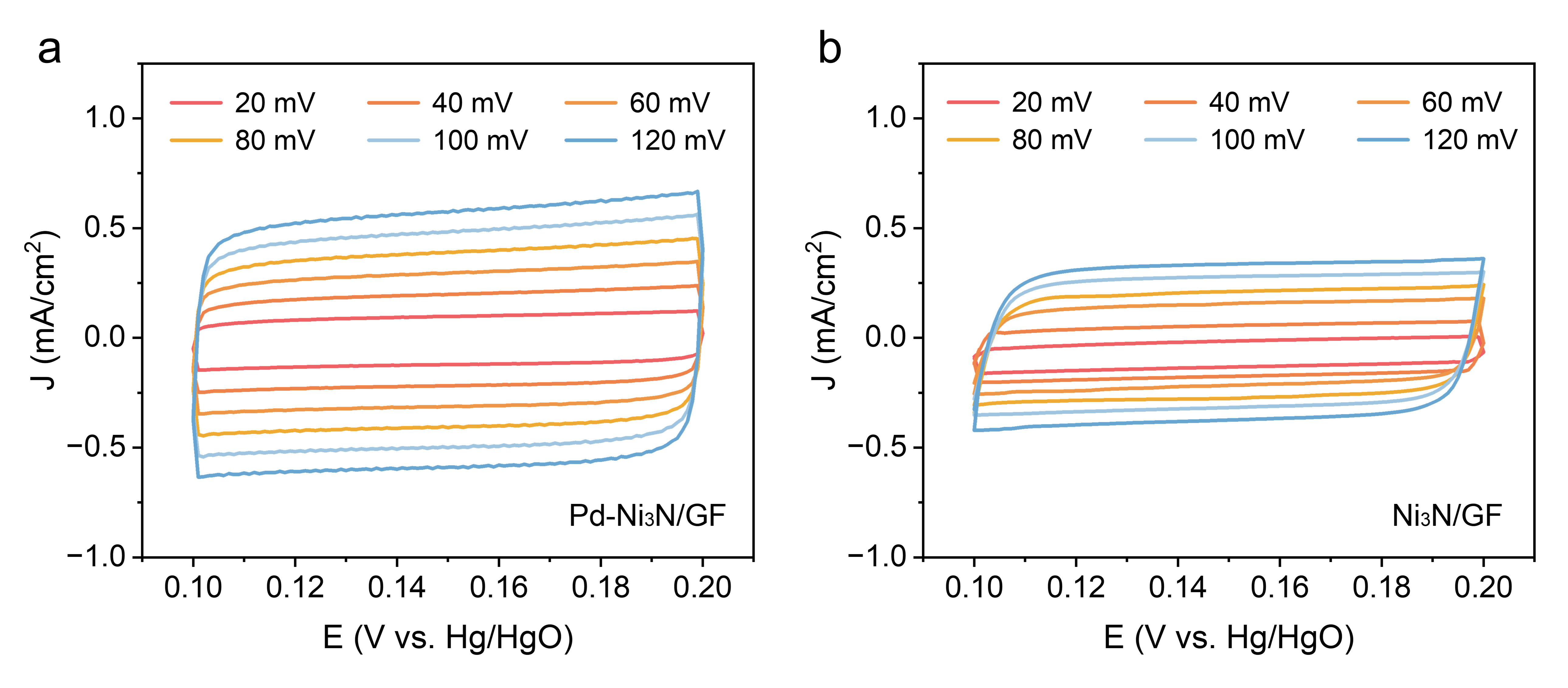


**Figure S11.** CVs curves at various scan rates of (a) Pd-Ni_3_N/GF and (b) Ni_3_N/GF are taken in a potential window (0.1-0.2 V vs. Hg/HgO) at various scan rates of 20, 40, 60, 80, 100, and 120 mV/s in 1 M K_2_CO_3_ electrolyte.


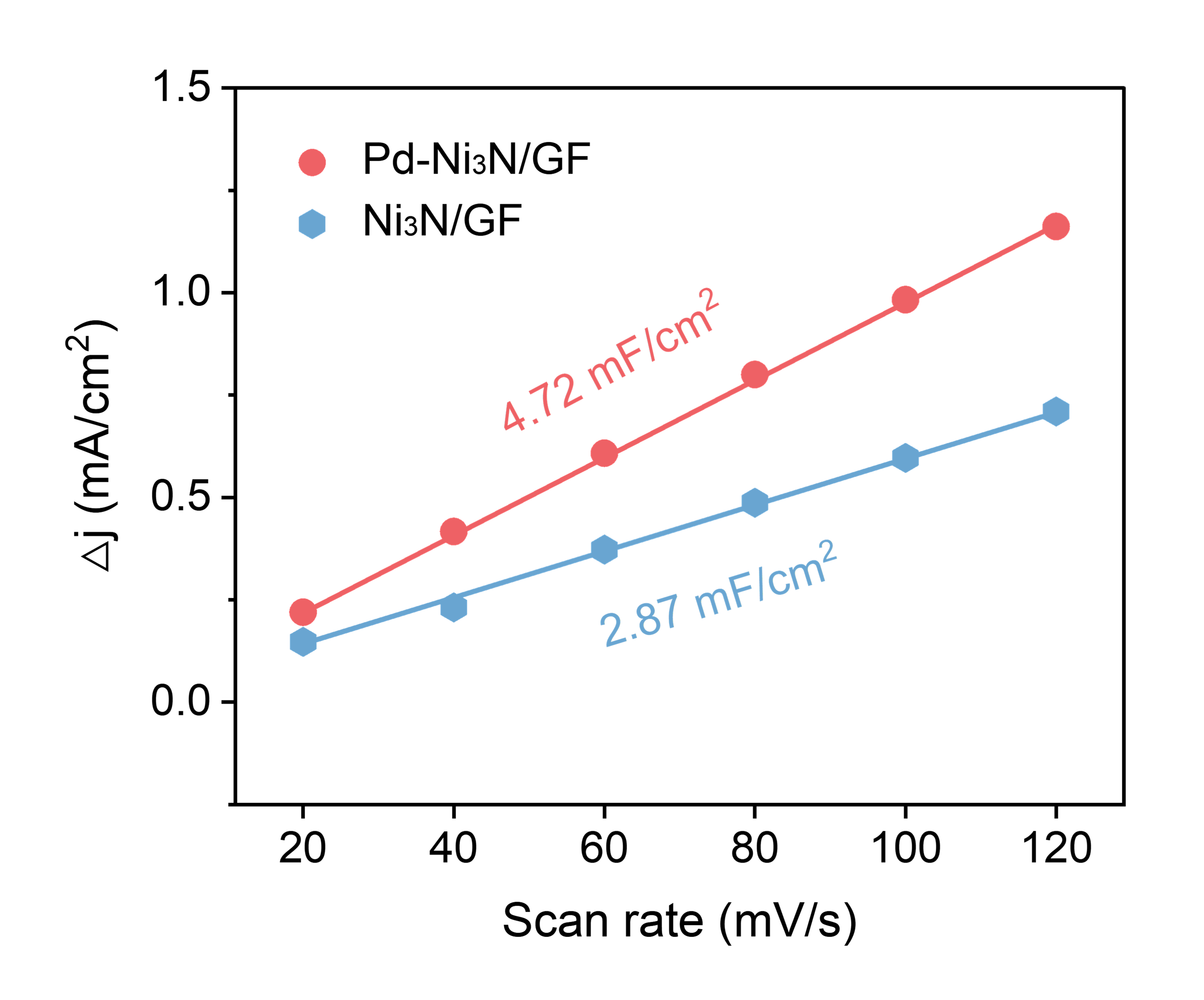


**Figure S12.** The capacitive current densities of the Pd-Ni_3_N/GF and Ni_3_N/GF.


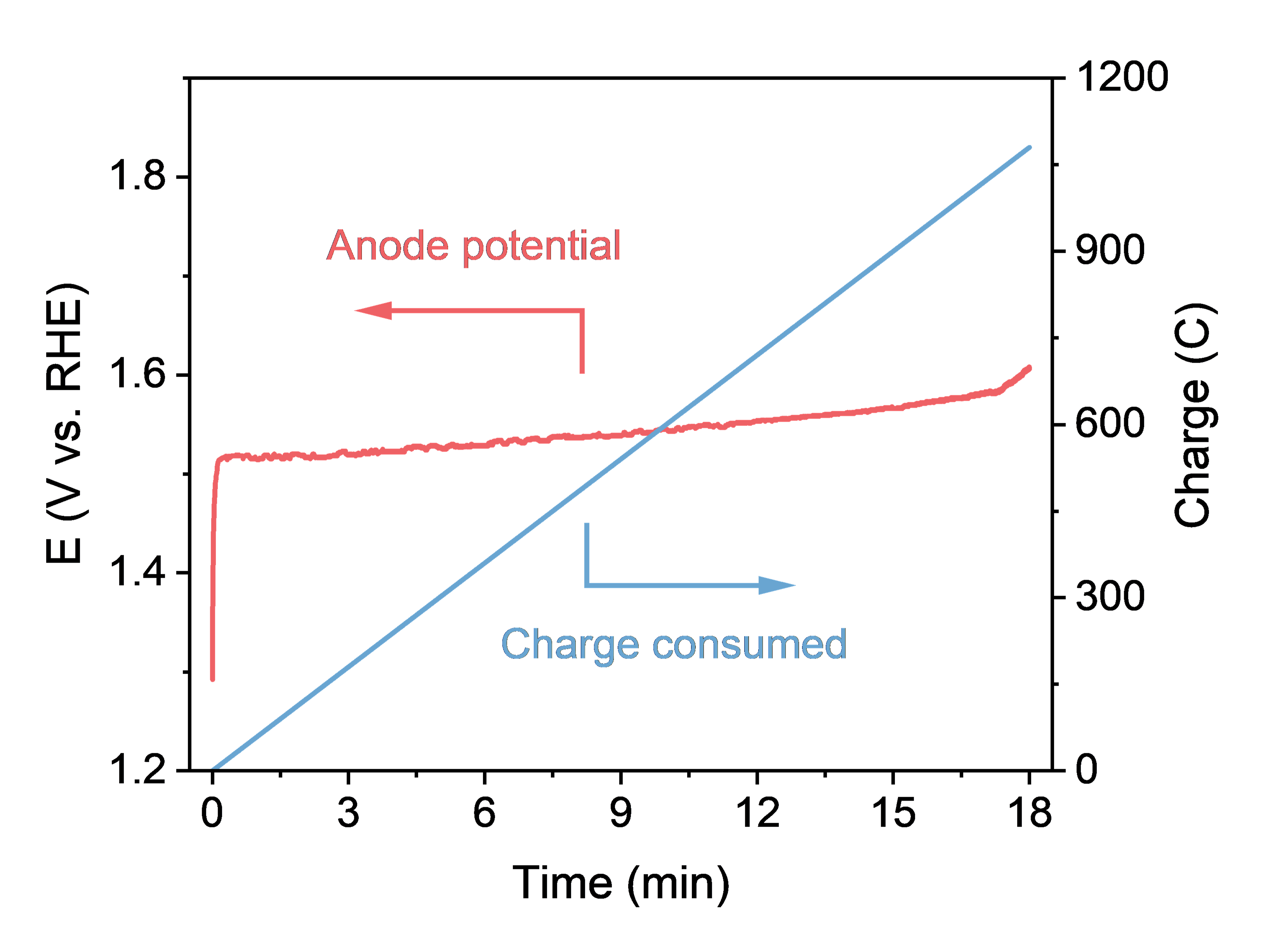


**Figure S13.** Anode potential and charge passed during **1a** electrooxidation in flow electrolyzer.


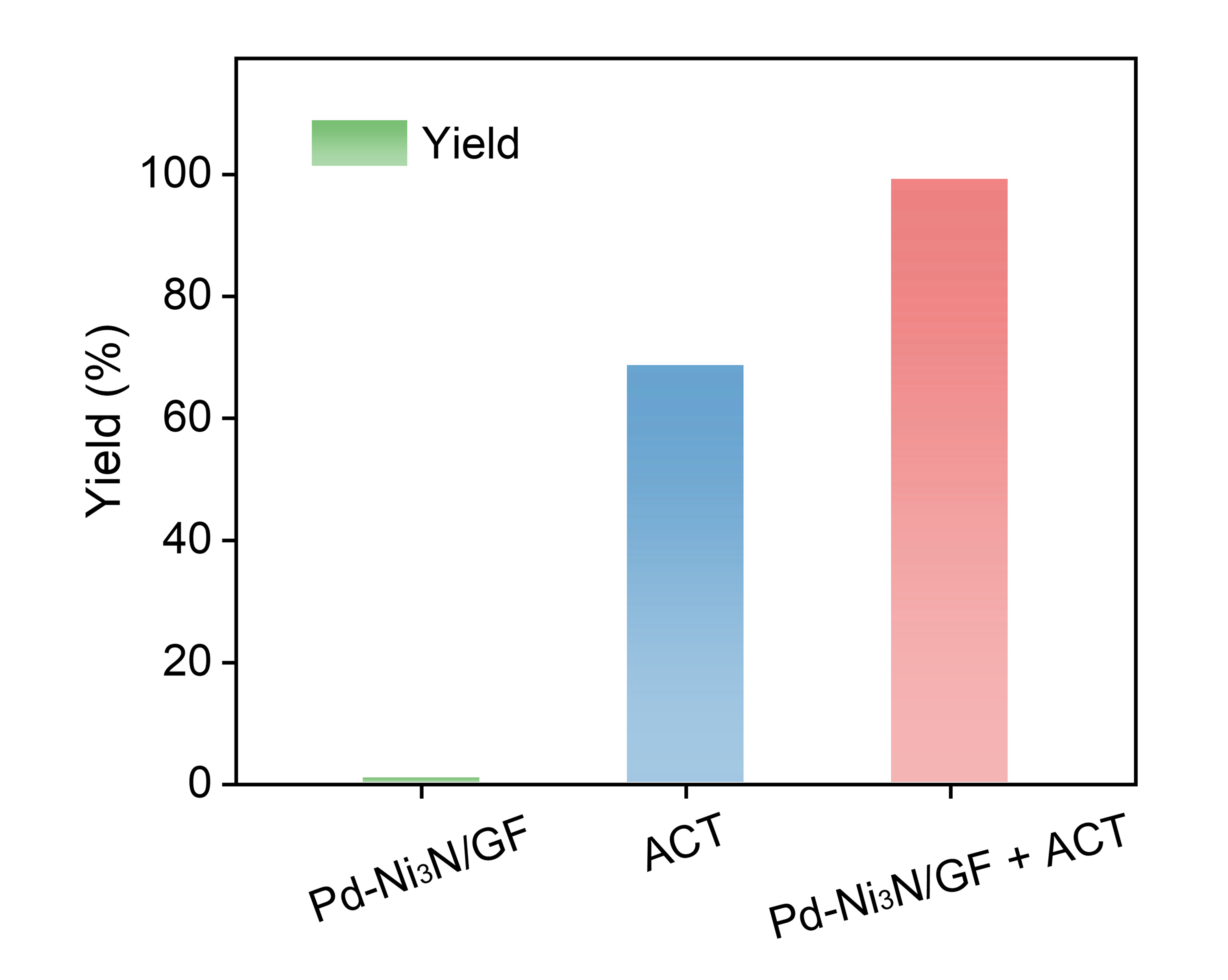


**Figure S14.** Yield (%) of **1b** for Pd-Ni_3_N/GF, ACT and Pd-Ni_3_N/GF + ACT during **1a** electrooxidation in flow electrolyzer. In the ACT system, a bare GF electrode is used as the working electrode.


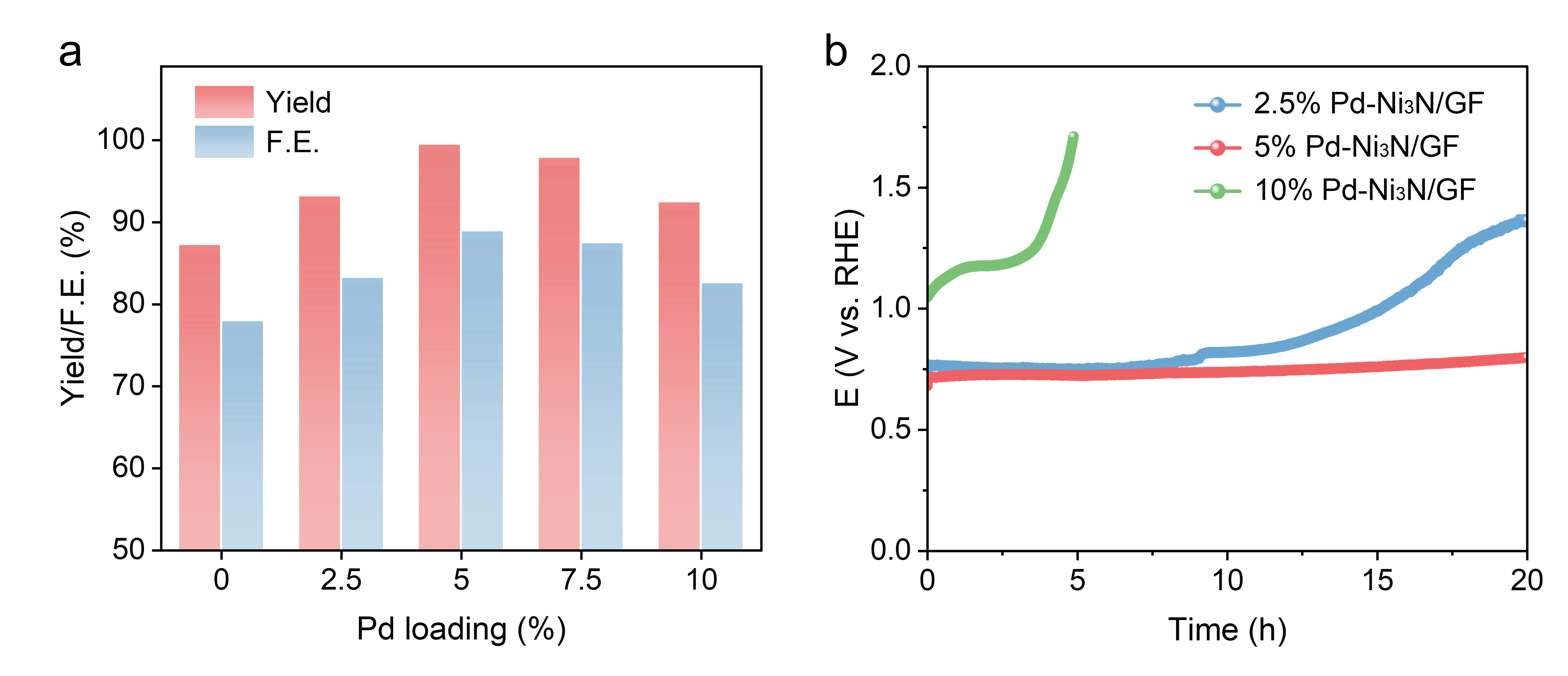


**Figure S15.** (a) Yield (%) and F.E. (%) of **1b** at different Pd loading of Pd-Ni_3_N/GF during **1a** electrooxidation in flow electrolyzer. (b) Chronopotentiometry testing of 2.5% Pd-Ni_3_N/GF, 5% Pd-Ni_3_N/GF, and 10% Pd-Ni_3_N/GF in 1 M K_2_CO_3_ electrolyte in the presence of **1a** and ACT in batch reactor.


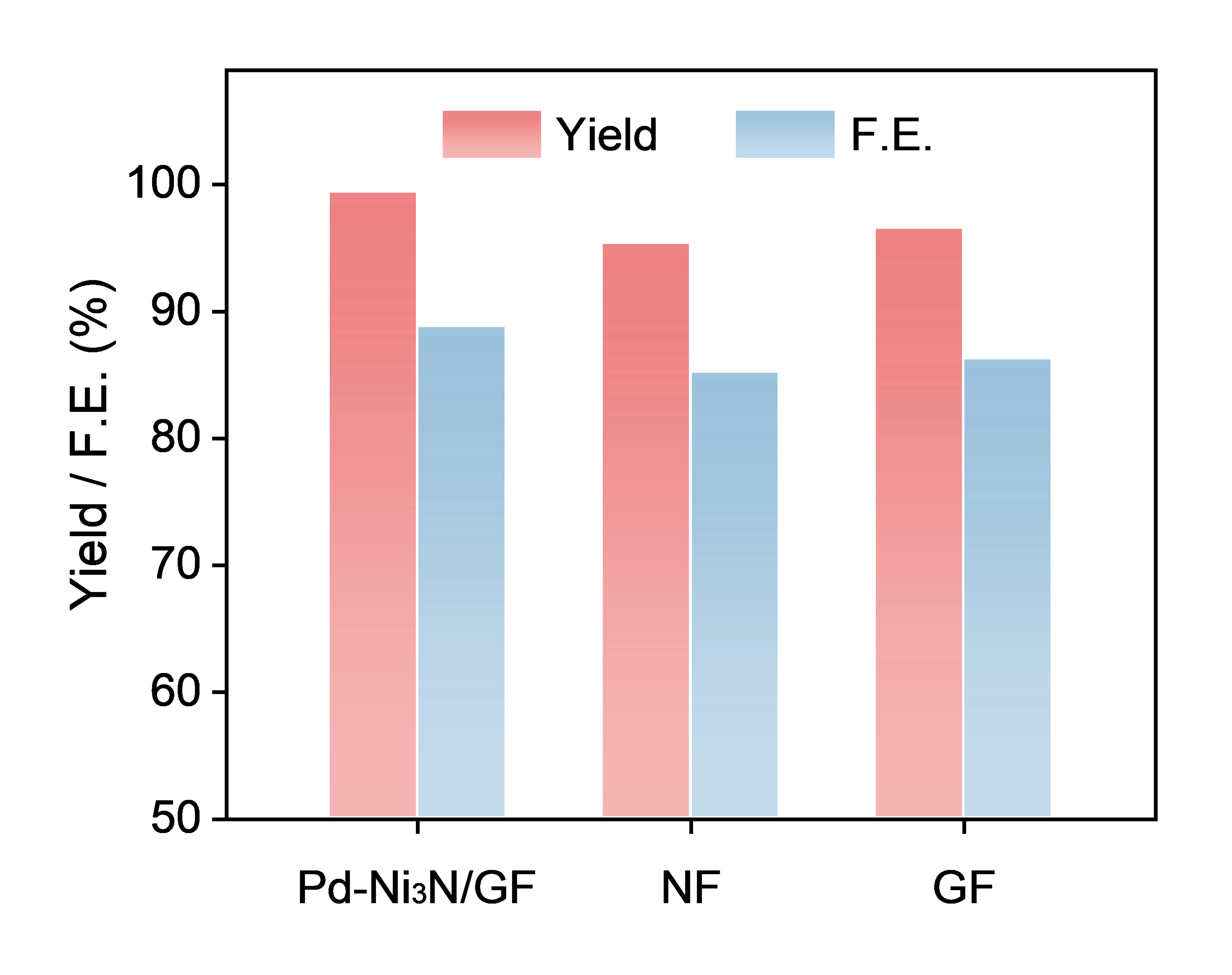


**Figure S16.** Yield (%) and F.E. (%) of **1b** for different cathode electrocatalysts of Pd-Ni_3_N/GF, NF, and GF during **1a** electrooxidation in flow electrolyzer.


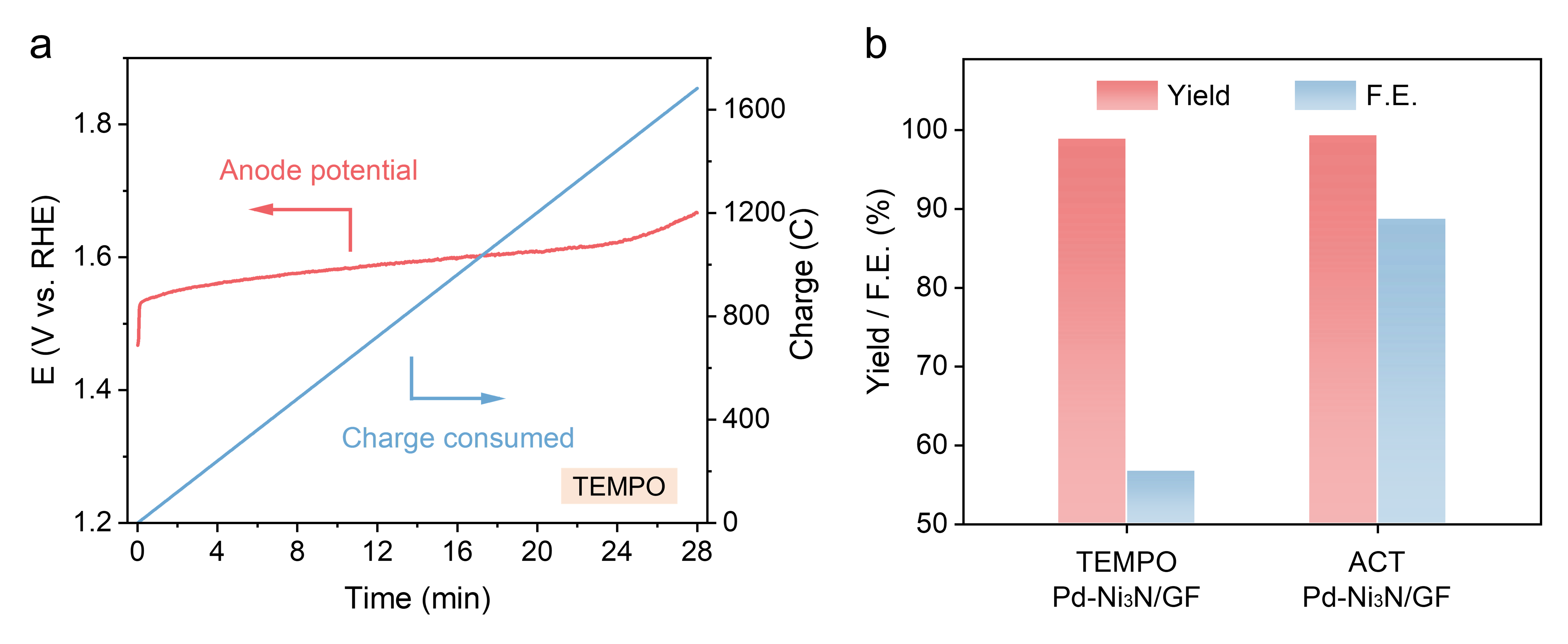


**Figure S17.** (a) Anode potential and charge passed of constant-current electrolysis of **1a** using TEMPO. (b) Comparison yield and F.E. for TEMPO and ACT during **1a** electrooxidation in the flow electrolyzer.


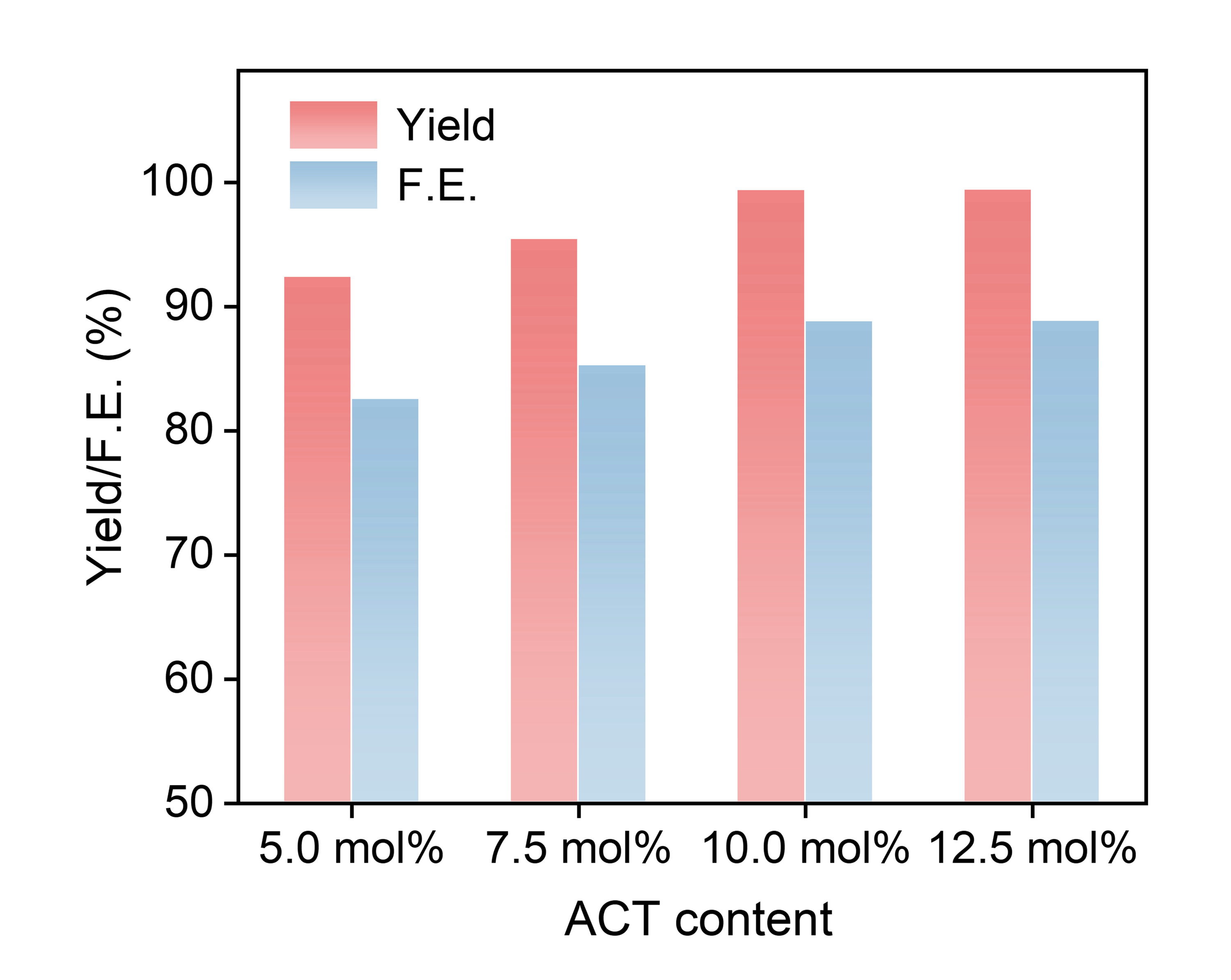


**Figure S18.** Yield (%) and F.E. (%) of **1b** at different dosage of ACT during **1a** electrooxidation in flow electrolyzer.


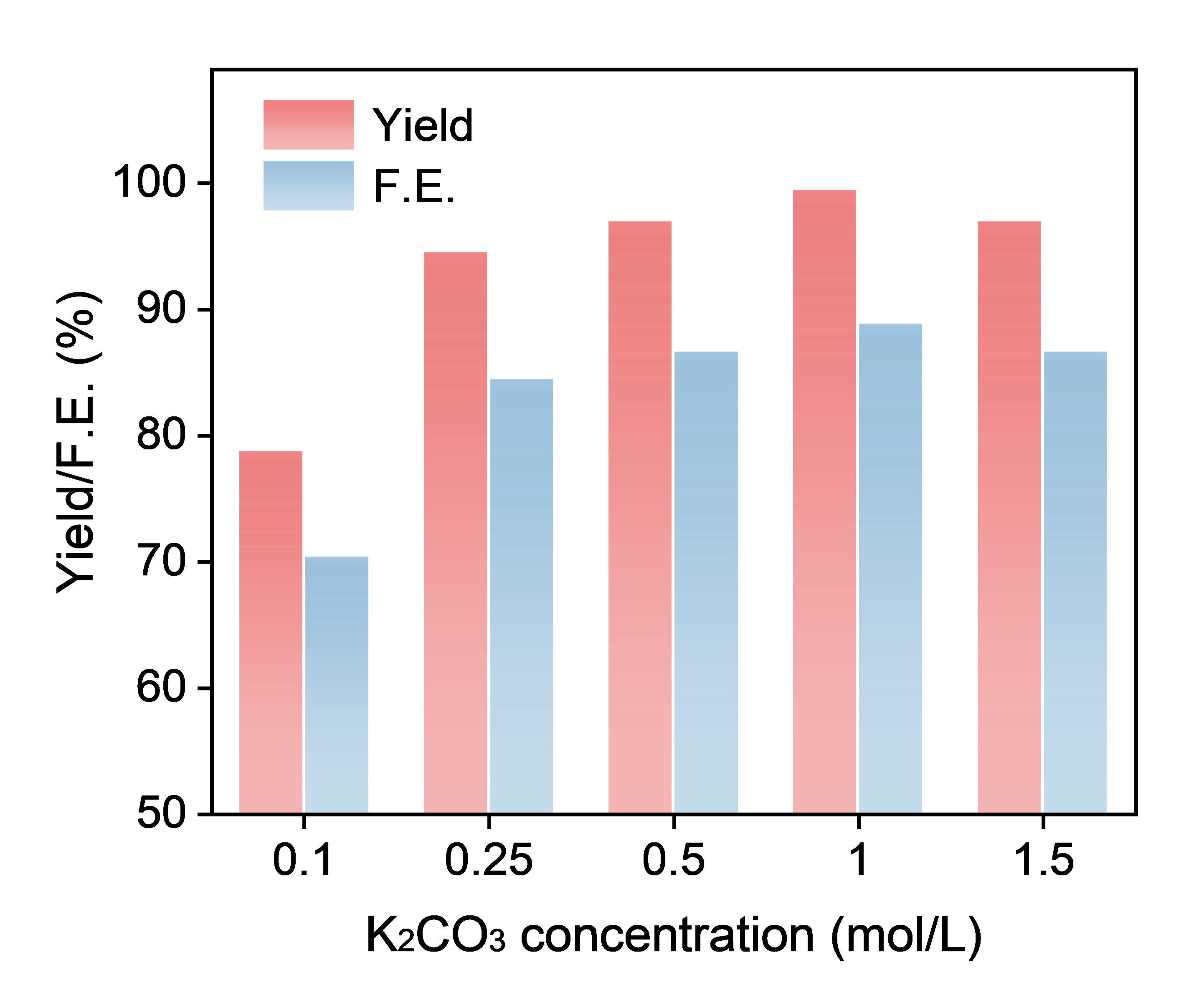


**Figure S19.** Yield (%) and F.E. (%) of **1b** at different concentration of K_2_CO_3_ during **1a** electrooxidation in flow electrolyzer.


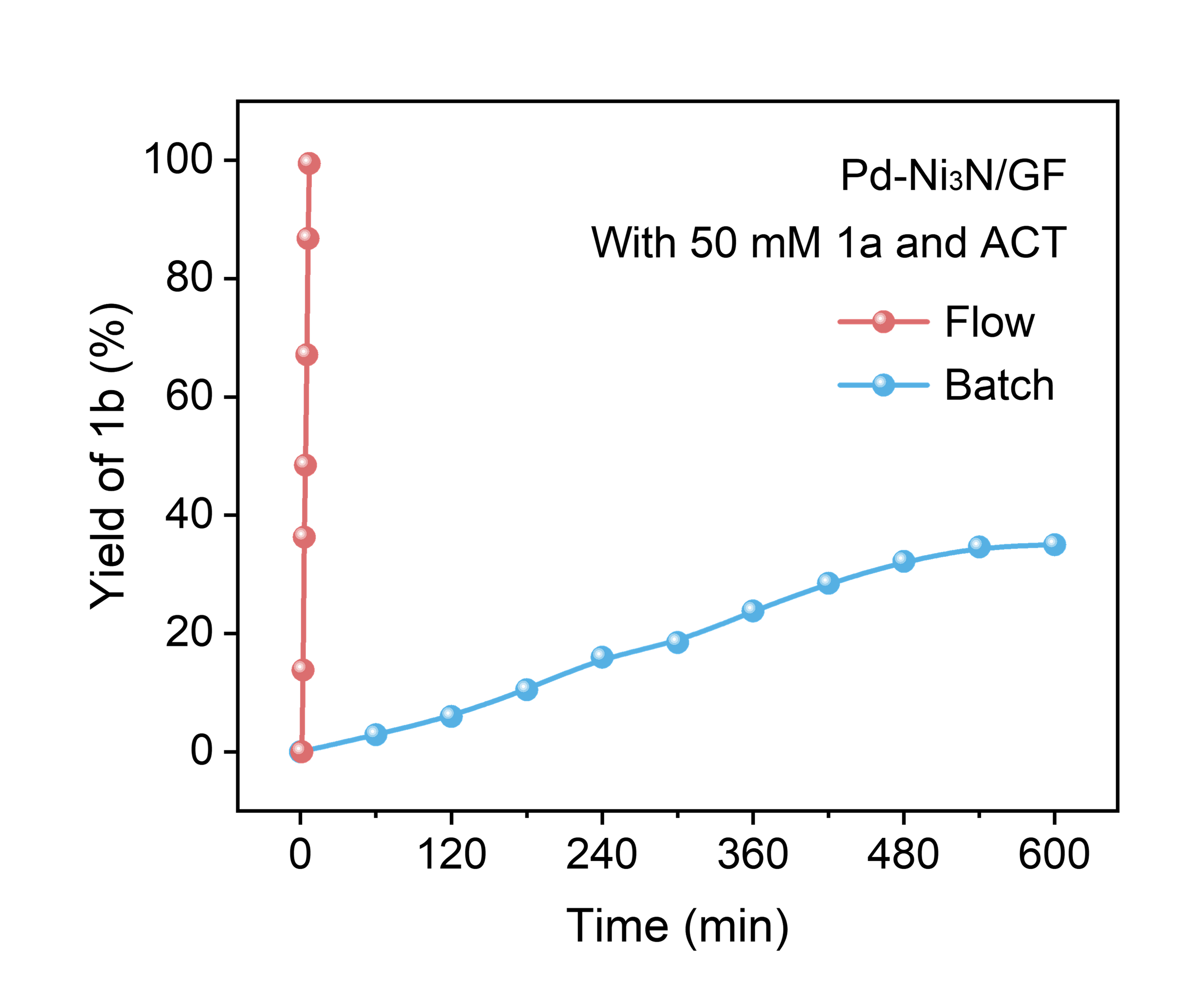


**Figure S20.** Yield (%) of **1b** during **1a** electrooxidation in batch reactor and flow electrolyzer.


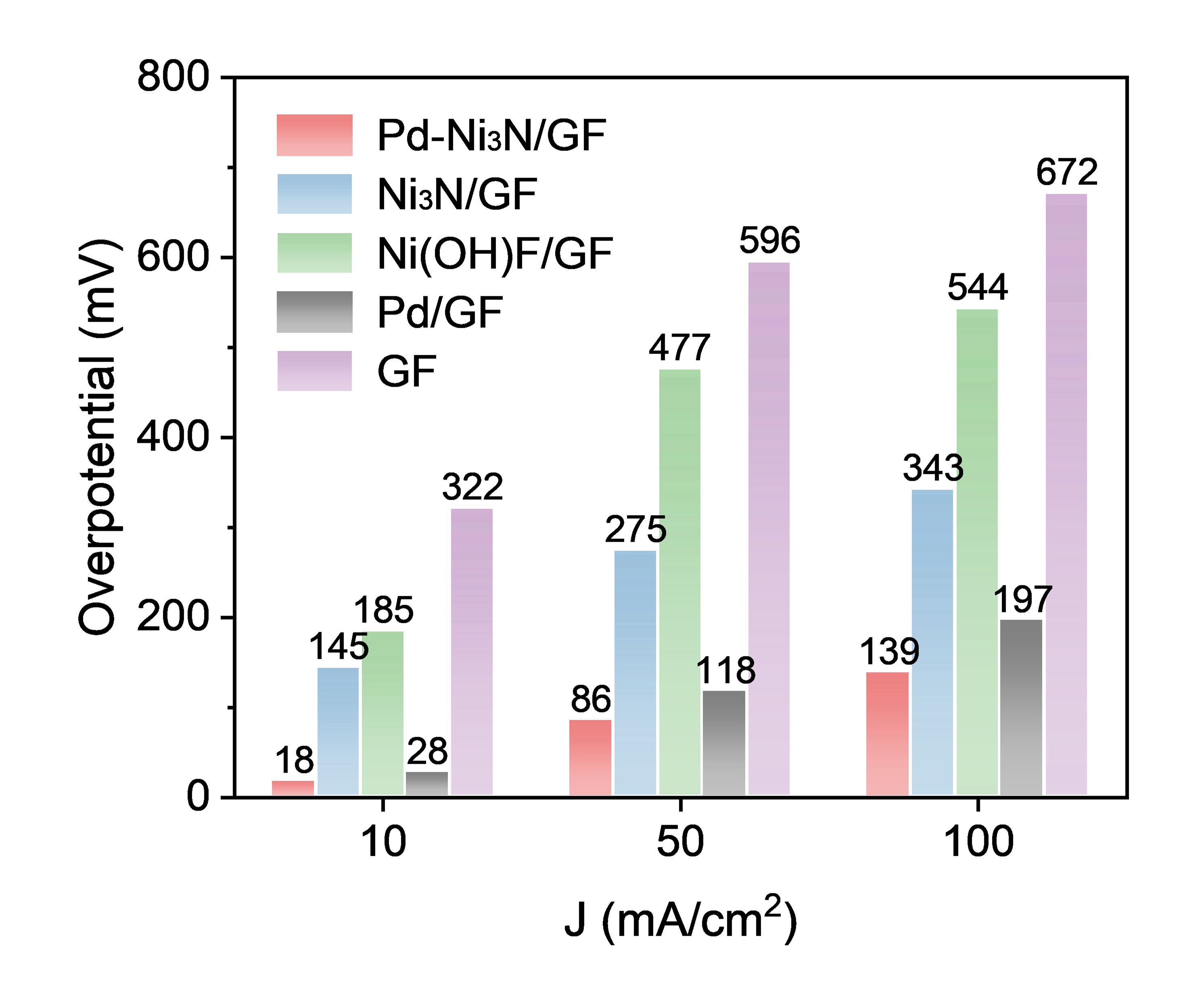


**Figure S21.** Comparison of HER overpotential of different electrocatalysts at selected current densities.


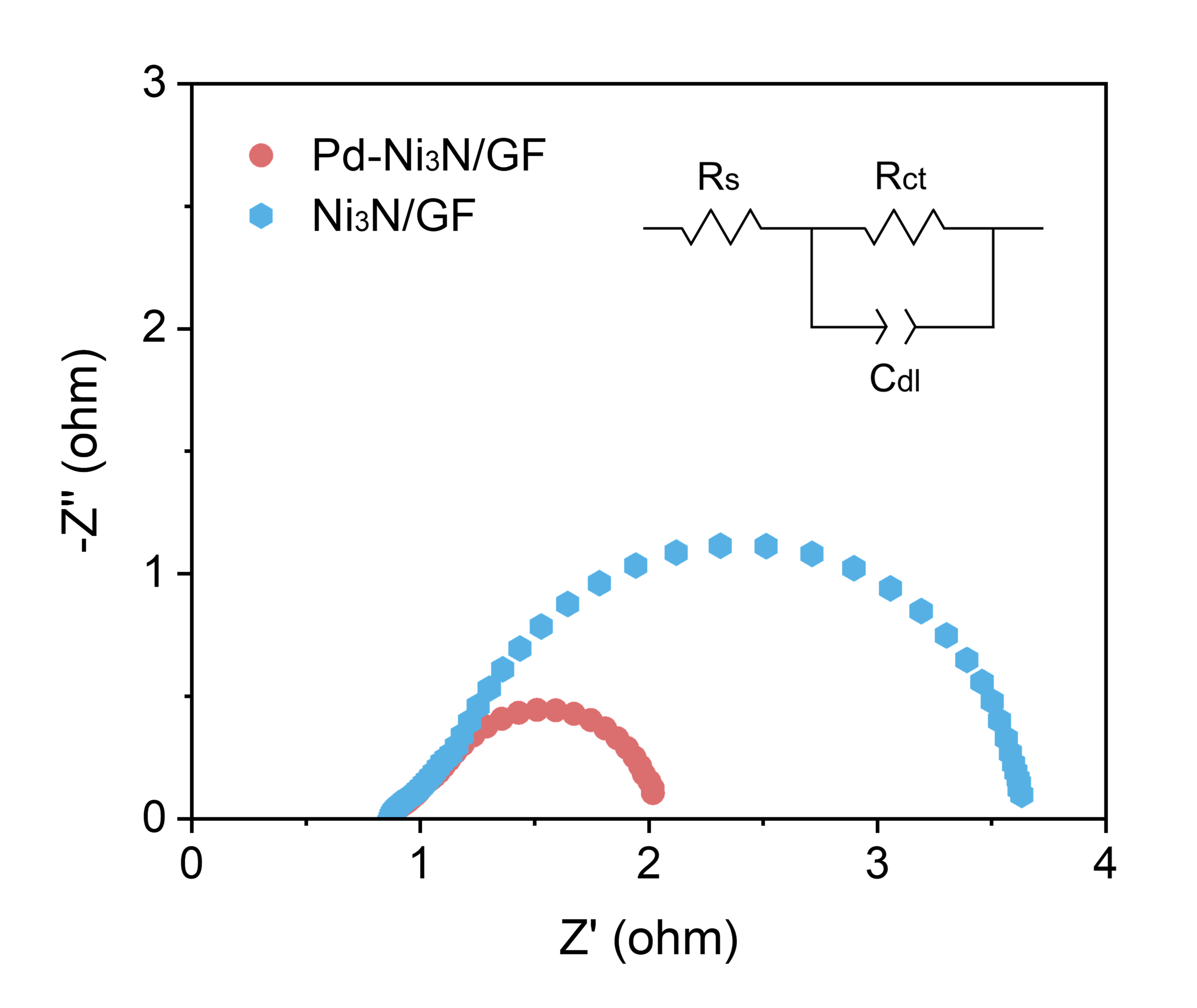


**Figure S22.** Nyquist plots of the Pd-Ni_3_N/GF and Ni_3_N/GF catalysts, the inset shows the corresponding equivalent circuit diagram.


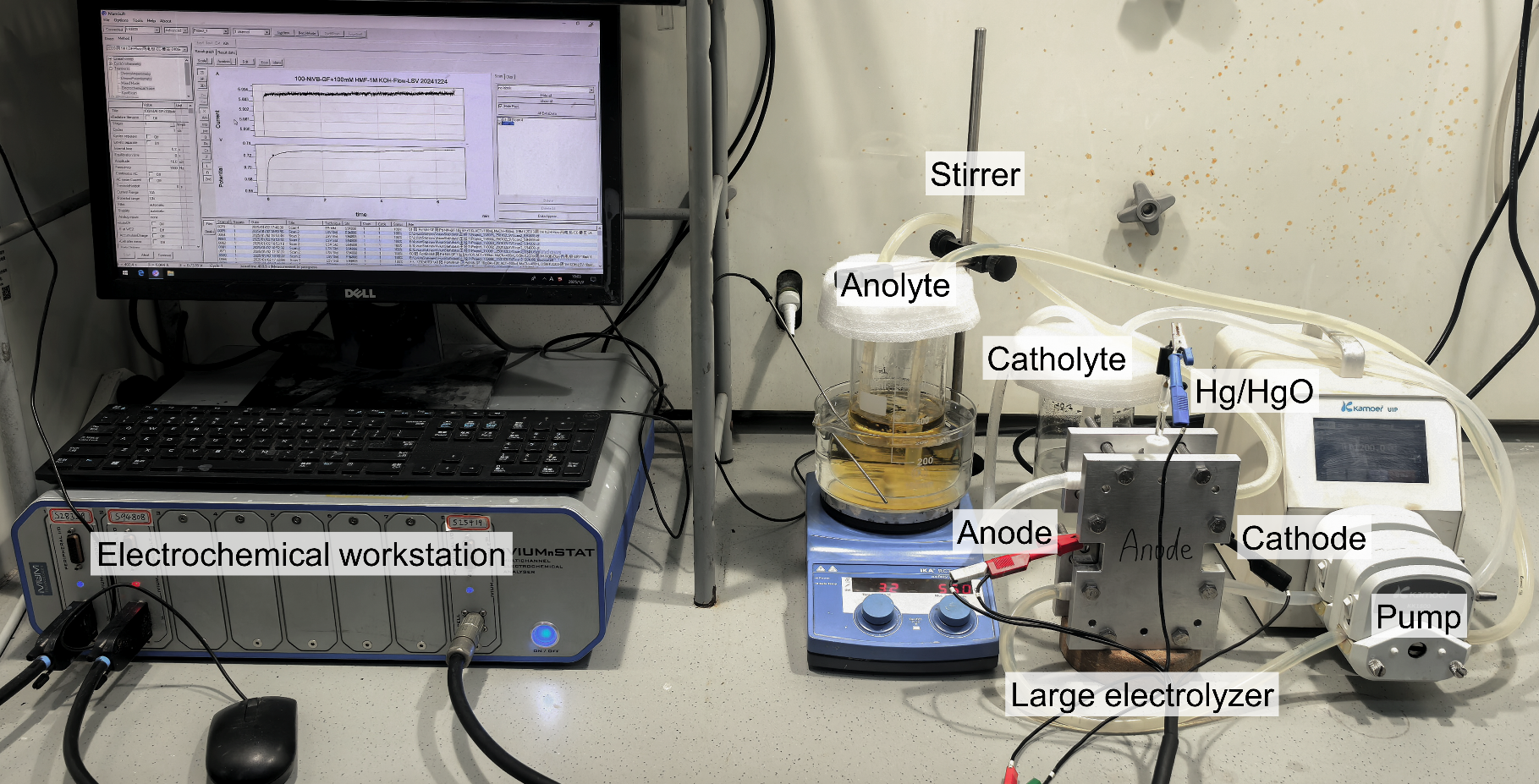


**Figure S23.** Electrolysis setup for simultaneous production of **1b** and H_2_ in large flow electrolyzer.


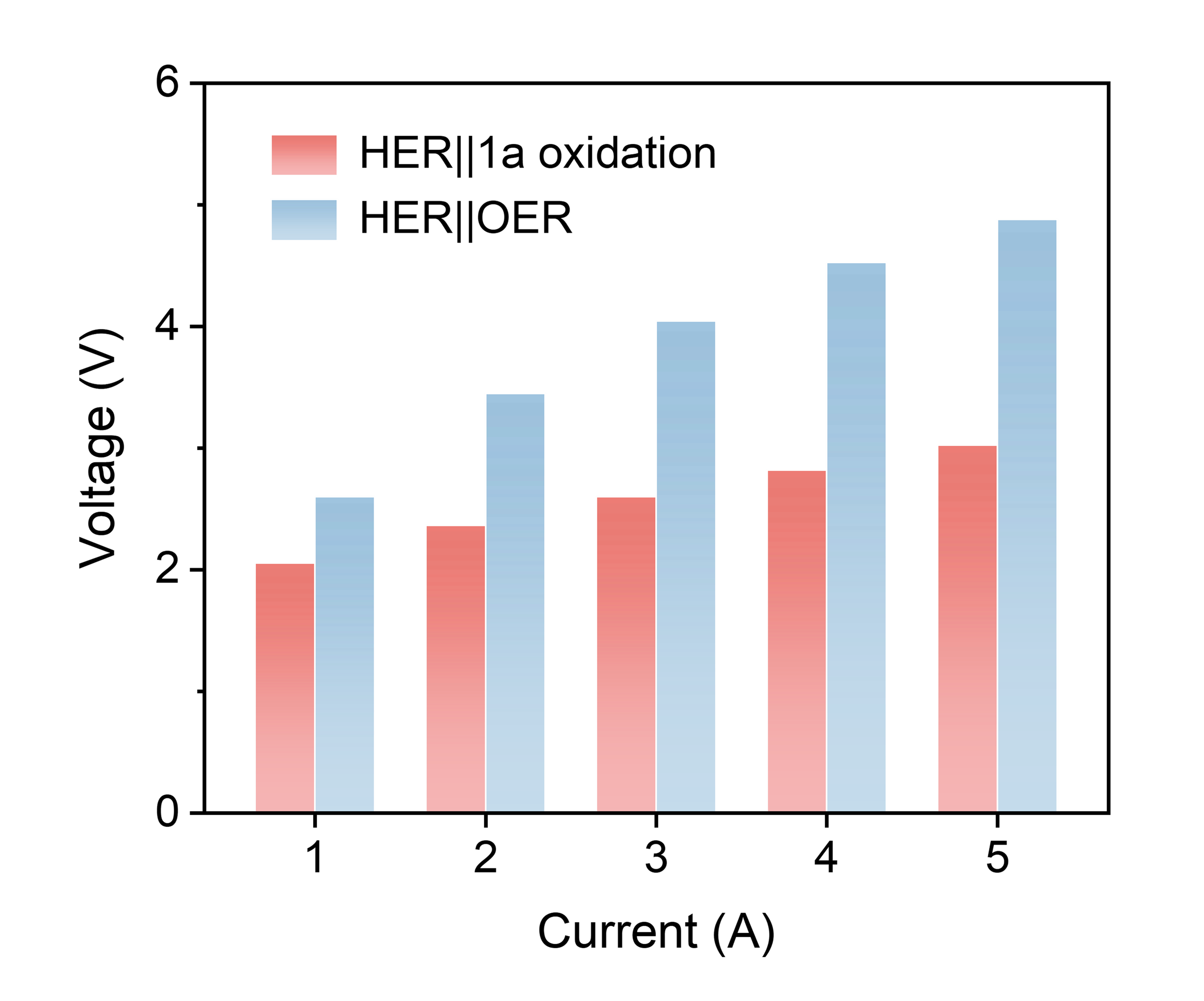


**Figure S24.** The voltage comparison at different current for HER||OER and HER||**1a** oxidation (**1a**, 15 g) in large flow electrolyzer.


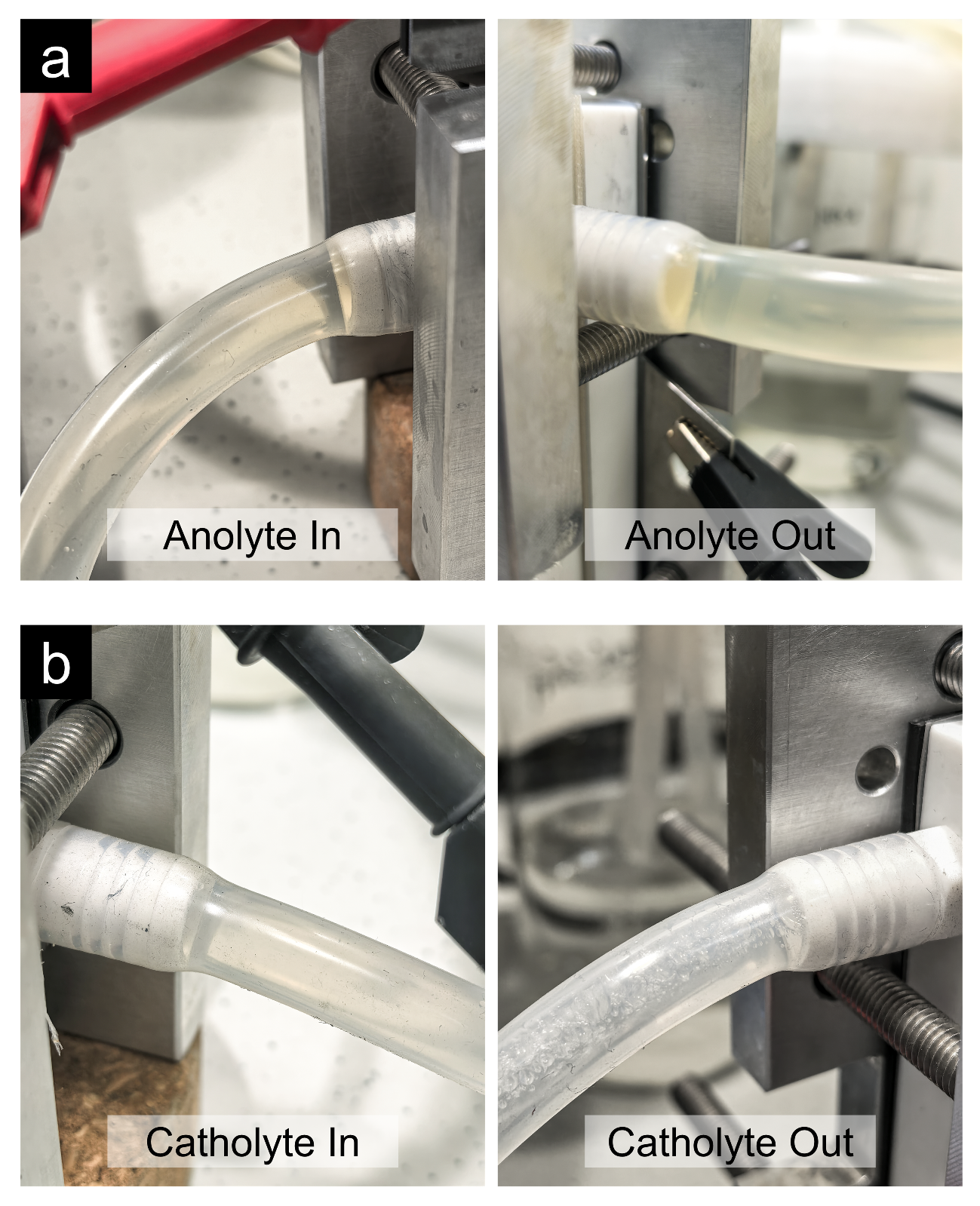


**Figure S25.** Comparison (a) anolyte and (b) catholyte inlet and outlet during **1a** electrooxidation in large flow electrolyzer.


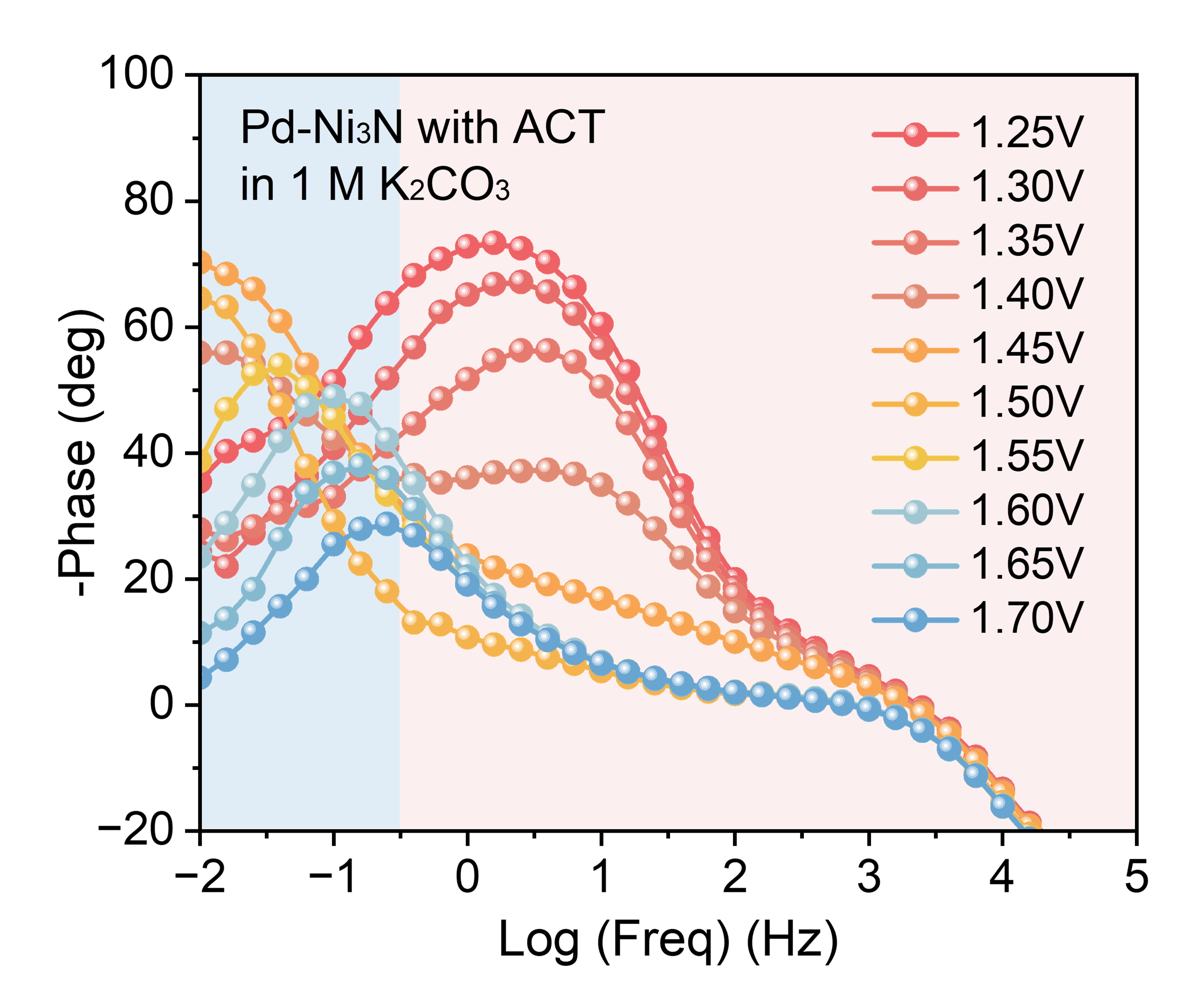


**Figure S26.** Bode plots of Pd-Ni_3_N/GF at various voltages (increased from 1.25-1.70 V vs. RHE) in 1 M K_2_CO_3_ with ACT.


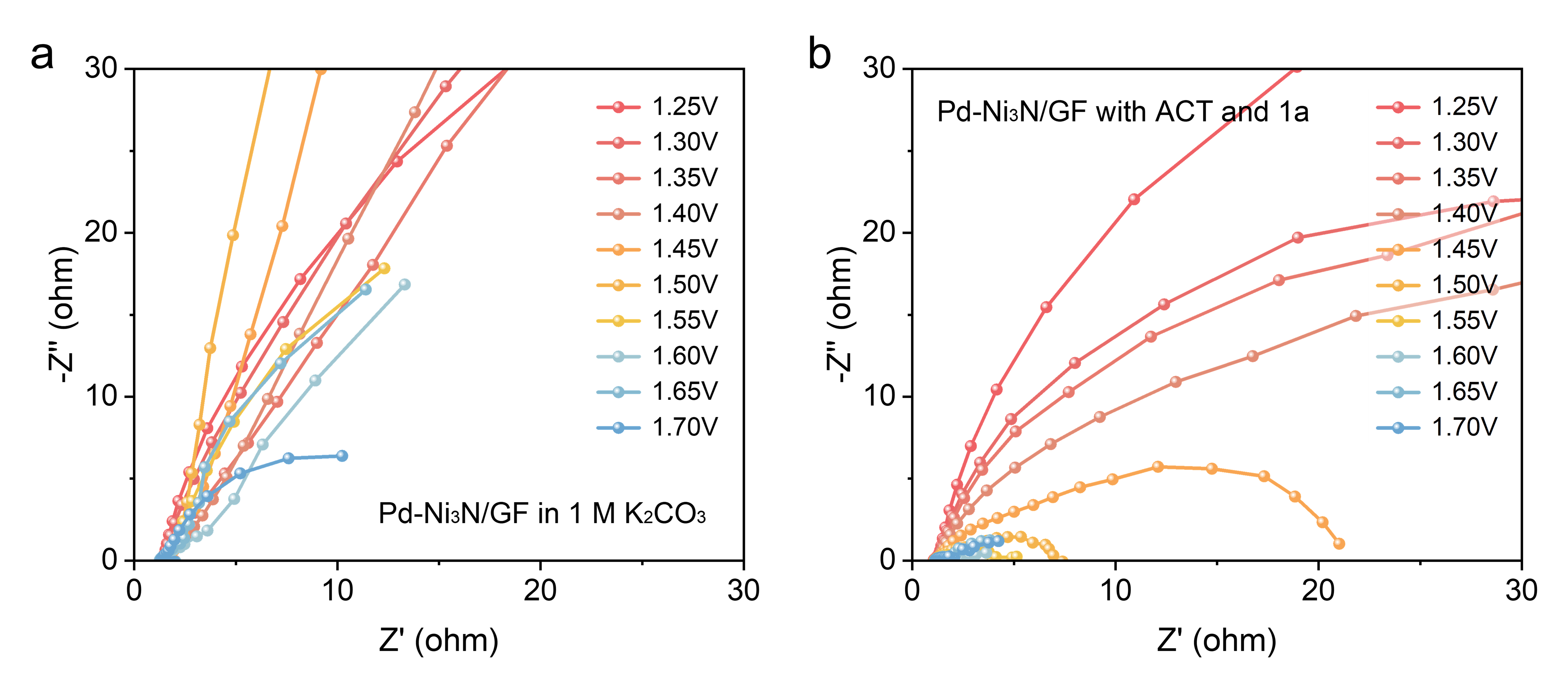


**Figure S27.** Nyquist plots of Pd-Ni_3_N/GF at various voltages (increased from 1.25-1.70 V vs. RHE) in (a) 1 M K_2_CO_3_ and (b) with ACT + **1a**.


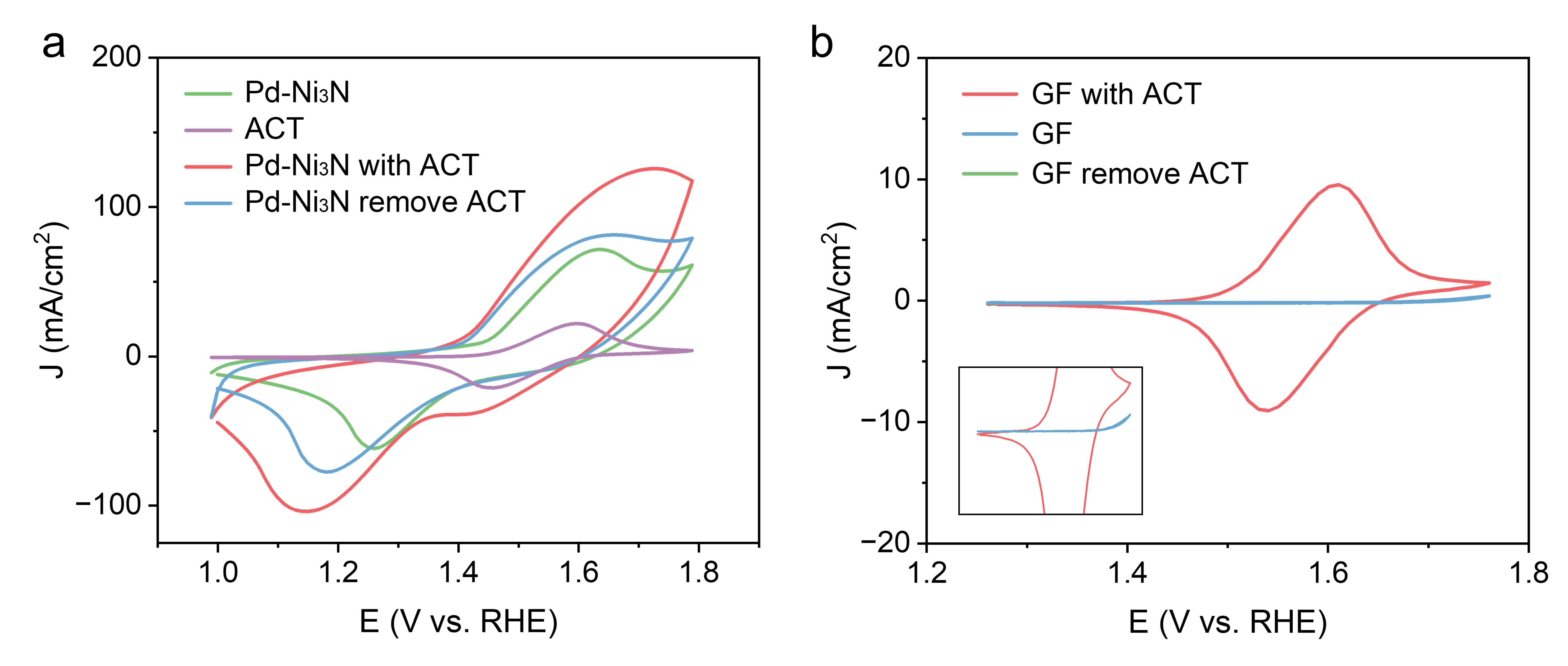


**Figure S28.** (a) CV curves of Pd-Ni_3_N/GF, ACT, Pd-Ni_3_N/GF with ACT and the corresponding electrodes remove ACT in batch reactor. (b) CV curves of GF as a working electrode without, with and the corresponding GF remove ACT in 1 M K_2_CO_3_ electrolyte in batch reactor.


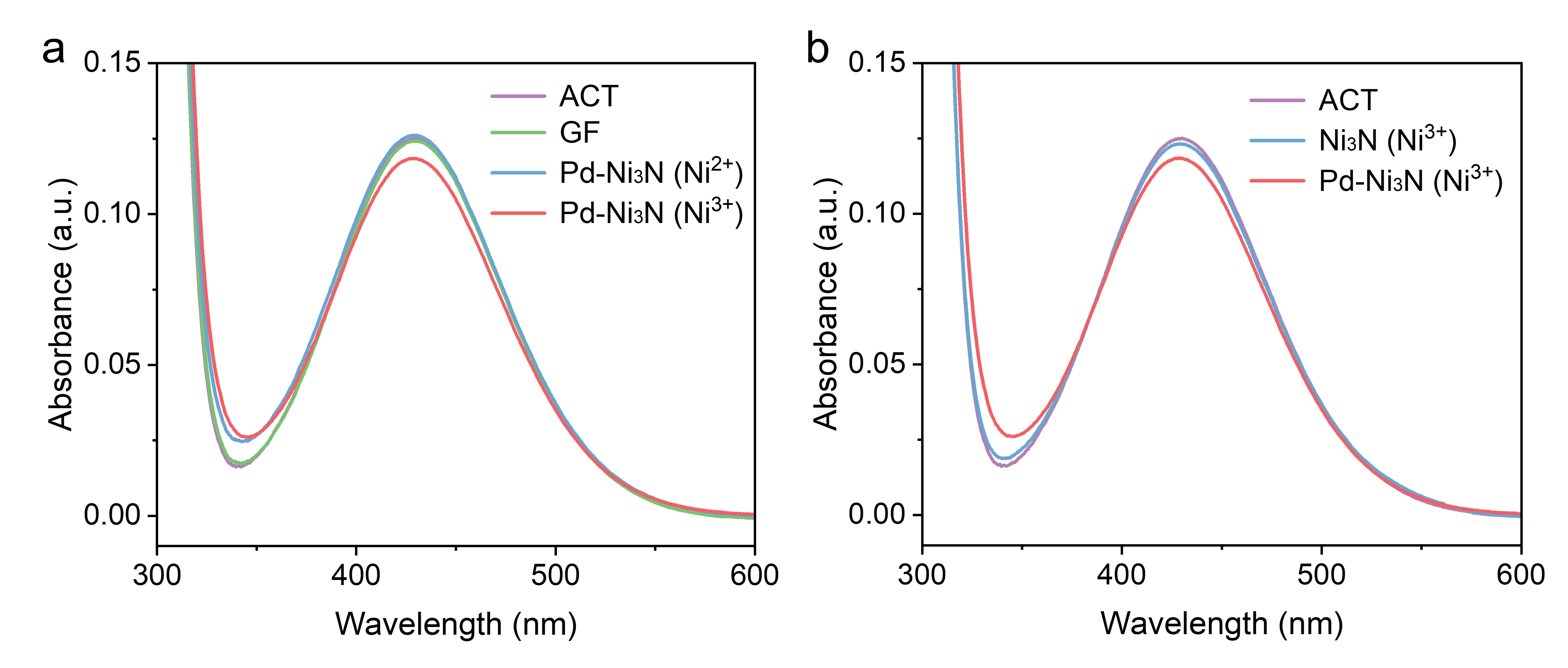


**Figure S29.** (a) UV-vis absorption of ACT adsorption spectra for GF, Pd-Ni_3_N/GF (Ni^2+^) and Pd-Ni_3_N/GF (Ni^3+^) electrodes. (b) UV-vis absorption of ACT adsorption spectra for Pd-Ni_3_N/GF (Ni^3+^) and Ni_3_N/GF (Ni^3+^) electrodes.


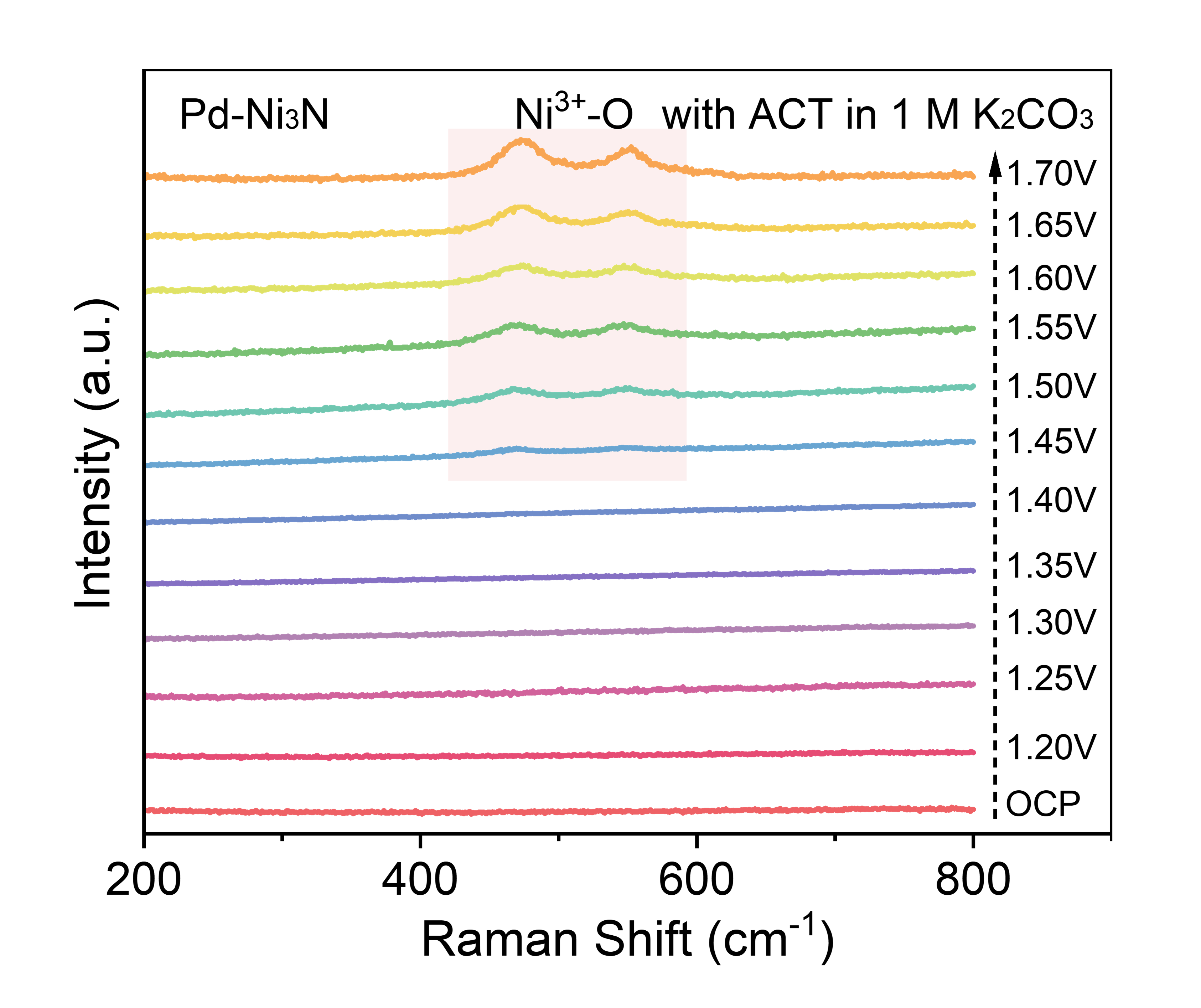


**Figure S30.** In situ Raman spectra taken on the Pd-Ni_3_N surface in 1 M K_2_CO_3_ with ACT at various voltages (increased from 1.20-1.70 V vs. RHE).


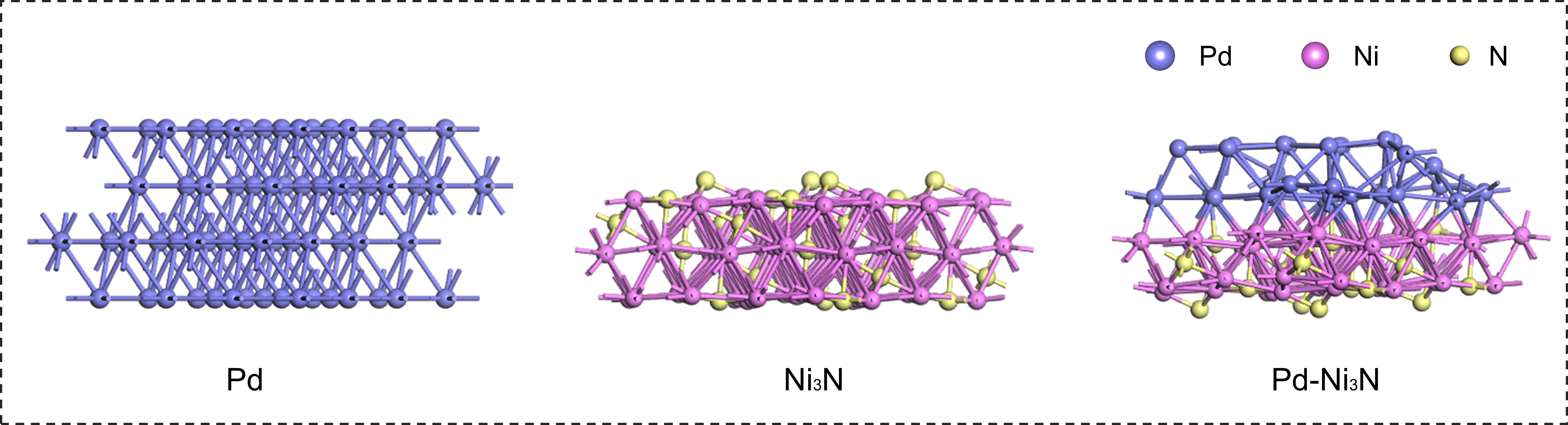


**Figure S31.** The theoretical models of Pd, Ni_3_N, and Pd-Ni_3_N.


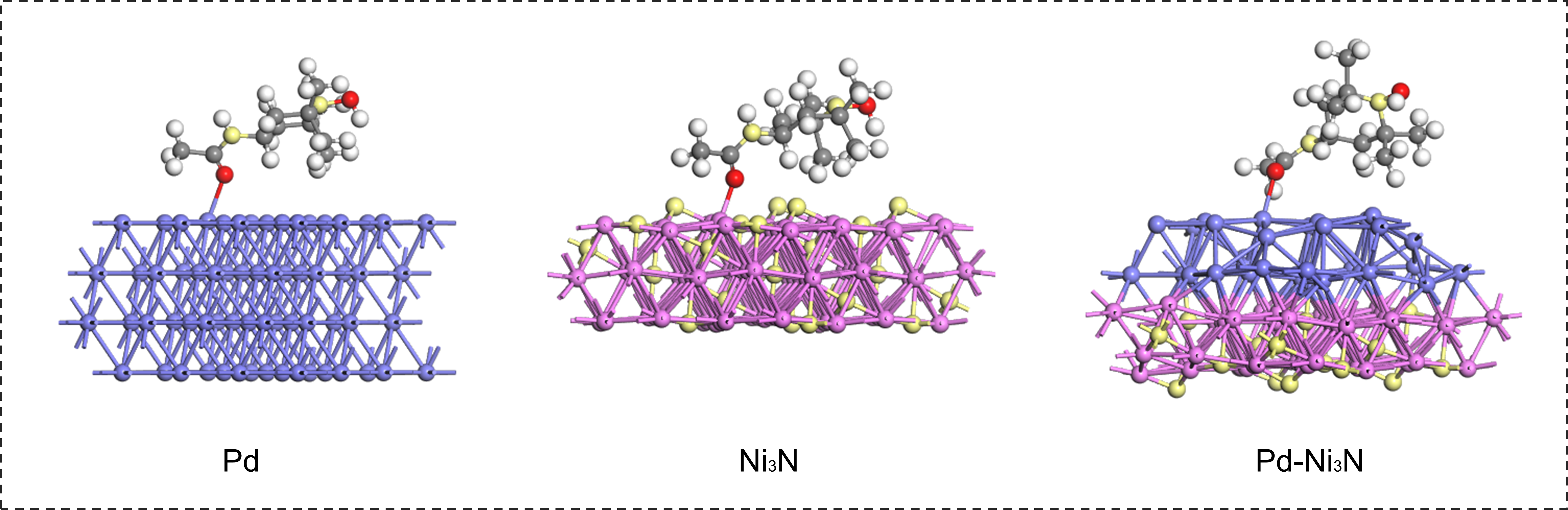


**Figure S32.** The adsorption structures of ACTH on Pd, Ni_3_N, and Pd-Ni_3_N.

**Table S1**. Comparison of the catalytic performance of representative state-of-the-art catalysts reported in the literatures and the Pd-Ni_3_N/ACT catalysts towards electrooxidation and HER.

| Catalyst | Electrooxidation performance | | | | HER performance | | | Electrooxidation//HER | | Ref. |
| --- | --- | --- | --- | --- | --- | --- | --- | --- | --- | --- |
|  | Substrate  Concentration  Electrolyte | Potential [V_RHE_] at current density  [mA/cm^2^] | Faradaic efficiency  (%) | Yield  (%) | Electrolyte | Overpotential at current density  [mA/cm^2^] | Stability  (h) | Product  Productivity  (mmol/h) | H_2_  Productivity  (mmol/h) |  |
| **Pd-Ni_3_N/GF** | **19-hydroxyandrost-4-ene-3,17-dione**  **(50 mM)**  **1 M K_2_CO_3_** | **1.50**  **(279)** | **88** | **98** | **1 M KOH** | **18(-10)**  **139(-100)** | **100** | **70.9** | **93.1** | This work |
| MoNi_4_@MoO_2_ | Cyclohexanone  (400 mM)  1 M KOH | 1.40  (200) | 96 | - | 1 M KOH | 68(-100) | 200 | 0.6 | - | [5] |
| Mo-Ni_2_P@Ni_12_P_5_-VP/NF | Cyclohexanol  (100 mM)  1 M KOH | 1.38  (100) | - | 91.4 | 1 M KOH | 140 (-300) | 600 | - | - | [6] |
| Mo_0.8_Ni_0.2_N-Ni_3_N/NF | Benzylamine  (25 mM)  0.1 M KOH | 1.54  (240) | 99 | 99 | 0.1 M KOH | 49(-10) | 140 | - | - | [7] |
| Cu_x_Ni_2-x_P | Glycerol  (10 mM)  1 M KOH | 1.54  (215) | 79.3 | - | 1 M KOH | - | - | 2.05 | 3.8 | [8] |
| Co_2_P-Ni_2_P/CC | Ethylene glycol  (500 mM)  1 M KOH | 1.349  (200) | 90 | - | 1 M KOH | 56(-10) | 40 | - | - | [9] |
| Ru-SA/NSC | Benzyl alcohol  (16.7 mM)  1 M KOH | 0.97  (10) | 100 | 96 | 1 M KOH | 39(-10) | 30 | - | - | [10] |
| Mo-Ni_2_P/Ni_12_P_5_@NF | Benzyl alcohol  (100 mM)  1 M KOH | 1.304  (100) | 96.5 | 94.1 | 1 M KOH | 130(-10) | 12 | - | - | [11] |
| Cr-NiO/ACT | 19-hydroxyandrost-4-ene-3,17-dione  (100 mM)  1 M Na_2_CO_3_ | 1.39  (100) | 91 | 97 | 1 M KOH | 35(-10) | 100 | 81.4 | 81.2 | [1c] |
| Rh-SA/NiFe NMLDH | HMF  (10 mM)  1 M KOH | 1.30  (50) | 98.5 | - | 1 M KOH | 51(-10) | 50 | - | - | [12] |
| Pt_SA_-NiCo LDH/NF | Glycerol  (100 mM)  1 M KOH | 1.298  (100) | 88.7 | - | 1 M KOH | 63(-100) | 50 | - | - | [13] |
| NiVRu-LDHs NAs/NF | Glycerol  (100 mM)  1 M KOH | 1.24  (10) | 97 | - | 1 M KOH | 35(-10) | 50 | 12.5 | 17.9 | [14] |
| Au/Ni(OH)_2_ | Glycerol  (300 mM)  3 M KOH  Ethylene glycol  (300 mM)  3 M KOH | 0.95 (317.7)  1.15 (326.2) | 47  96 | - | 3 M KOH  3 M KOH | - | - | 56.9 (LA)  36.8 (GA) | 120.9  77.0 | [15] |
| Ni(OH)_2_-SDS/Ni foam | Cyclohexanone  (20 mM)  0.5 M KOH | 1.50  (17) | 93 | 86.5 | 0.5 M KOH | - | - | 0.20 | 14.9 | [16] |
| Au/CoOOH | benzyl alcohol  (100 mM)  1 M KOH | 1.50  (540) | 98 | 99 | 3 M KOH | - | - | 36.6 | 84.8 | [17] |
| Co@NPC-800 | Glucose  (100 mM)  1 M KOH | 1.46  (10) | - | 45.4 | 1 M KOH | 274(-10) | 15 | - | - | [18] |
| CoNiP-NIE | HMF  (10 mM)  1 M KOH | 1.29  (20) | 81.9 | 85.8 | 1 M KOH | 107.56(-10) | - | - | - | [19] |
| CNs@CoPt | Glycerol  (10 mM)  1 M KOH | 1.52  (100) | 77 | - | 1 M KOH | 19.1(-10) | 11 | - | - | [20] |
| Co_2_(OH)_3_Cl/FeOOH@NF | Cyclohexanol  (2 mM)  1 M KOH | 1.46  (10) | 94 | - | 1 M KOH | 98(-10) | 50 | 35.1 | - | [21] |
| NC/Ni-Mo-N/NF | Glycerol  (100 mM)  1 M KOH | 1.16(10)  1.25(50) | 96.7 | - | 1 M KOH | 72(-10) | 12 | - | - | [22] |
| NiFeO_x_-NF | Glucose  (100 mM)  1 M KOH | 1.30  (87.6) | 87 | 83 | 1 M KOH | 40.6(-10) | 24 | - | - | [23] |
| MoO_2_-FeP@C | HMF  (10 mM)  1 M KOH | 1.359  (10) | 97.8 | - | 1 M KOH | 103(-10)  190(-100) | 24 | - | - | [24] |
| NiSe@NiO_x_ | HMF  (10 mM)  1 M KOH | 1.36  (240) | 99 | 99 | 1 M KOH | 208(-10) | 16 | - | - | [25] |
| [Cu_x_S@Ni_0.75_Co_0.25_O_m_H](mailto:CuxS@Ni0.75Co0.25OmH)_n_ LDHs | HMF  (10 mM) | 1.3  (87) | 99 | 99 | 1 M KOH | 79(-10) | 20 | - | - | [26] |

**6. References**

[1] a) H. Wang, C. Li, J. An, Y. Zhuang, S. Tao, *J. Mater. Chem. A* **2021**, 9, 18421; b) S. Li, C. Li, K. Li, X. Sun, X. Zhong, J. He, Z. Xu, X. Liu, J. Zhang, F. Shao, M. Li, Z. Zhang, X. Li, J. Wang, *Chem. Eng. J.* **2022**, 446, 136659; c) S. Li, S. Wang, J. He, K. Li, Y. Xu, M. Wang, S. Zhao, Y. Wang, X. Li, X. Zhong, J. Wang, *Angew. Chem., Int. Ed.* **2023**, 62, e202306553; d) M. Wang, J. Li, S. Li, L. Liu, J. He, K. Li, Y. Xu, S. Zhao, W. Zhou, C. Li, X. Zhong, Z. Zhang, Z. Yao, J. Wang, *Chem. Eng. Sci.* **2024**, 285, 119589.

[2] J. P. Perdew, K. Burke, M. Ernzerhof, *Phys. Rev. Lett.* **1996**, 77, 3865.

[3] a) G. Kresse, J. Furthmüller, *Comp. Mater. Sci.* **1996**, 6, 15; b) G. Kresse, J. Furthmüller, *Phys. Rev. B* **1996**, 54, 11169.

[4] a) G. Kresse, D. Joubert, *Phys. Rev. B* **1999**, 59, 1758; b) P. E. Blöchl, *Phys. Rev. B* **1994**, 50, 17953.

[5] C. Lin, H. Lu, X. Ren, B. He, X. Peng, P. Chen, *Chem. Eng. J.* **2025**, 509, 161475.

[6] S. Fan, G. Yang, Y. Jiao, Y. Liu, J. Wang, H. Yan, H. Fu, *Adv. Mater.* **2025**, 37, 2502523.

[7] Y. Li, Y. Jiao, H. Yan, G. Yang, Y. Liu, C. Tian, A. Wu, H. Fu, *Angew. Chem., Int. Ed.* **2023**, 62, e202306640.

[8] L. Ma, Y. Miao, J. Yang, Y. Fu, Y. Yan, Z. Zhang, Z. Li, M. Shao, *Adv. Energy Mater.* **2024**, 14, 2401061.

[9] J. Zhang, X. Zhang, C. Shi, X. Yu, Y. Zhou, L. Di, *Small* **2024**, 20, 2406767.

[10] P. Zhu, Y. Shen, Z.-M. Zhang, D. Wang, B. Xi, X. An, S. Xiong, C. An, *ACS Catal.* **2024**, 14, 7674.

[11] J. Zhai, Z. Wang, X. Cao, Y. Zhao, Z. Lu, P. He, *Chem. Eng. J.* **2024**, 499, 156646.

[12] L. Zeng, Y. Chen, M. Sun, Q. Huang, K. Sun, J. Ma, J. Li, H. Tan, M. Li, Y. Pan, Y. Liu, M. Luo, B. Huang, S. Guo, *J. Am. Chem. Soc.* **2023**, 145, 17577.

[13] H. Yu, W. Wang, Q. Mao, K. Deng, Z. Wang, Y. Xu, X. Li, H. Wang, L. Wang, *Appl. Catal. B-Environ.* **2023**, 330, 122617.

[14] Q. Qian, X. He, Z. Li, Y. Chen, Y. Feng, M. Cheng, H. Zhang, W. Wang, C. Xiao, G. Zhang, Y. Xie, *Adv. Mater.* **2023**, 35, 2300935.

[15] Y. Yan, H. Zhou, S.-M. Xu, J. Yang, P. Hao, X. Cai, Y. Ren, M. Xu, X. Kong, M. Shao, Z. Li, H. Duan, *J. Am. Chem. Soc.* **2023**, 145, 6144.

[16] Z. Li, X. Li, H. Zhou, Y. Xu, S.-M. Xu, Y. Ren, Y. Yan, J. Yang, K. Ji, L. Li, M. Xu, M. Shao, X. Kong, X. Sun, H. Duan, *Nat. Commun.* **2022**, 13, 5009.

[17] Z. Li, Y. Yan, S.-M. Xu, H. Zhou, M. Xu, L. Ma, M. Shao, X. Kong, B. Wang, L. Zheng, H. Duan, *Nat. Commun.* **2022**, 13, 147.

[18] D. Li, Y. Huang, Z. Li, L. Zhong, C. Liu, X. Peng, *Chem. Eng. J.* **2022**, 430, 132783.

[19] Y. Song, W. Xie, Y. Song, H. Li, S. Li, S. Jiang, J. Y. Lee, M. Shao, *Appl. Catal. B-Environ.* **2022**, 312, 121400.

[20] S. Li, W. Xie, Y. Song, Y. Li, Y. Song, J. Li, M. Shao, *Chem. Eng. J.* **2022**, 437, 135473.

[21] M. Qin, R. Fan, J. Chen, H. Wang, X. Zheng, S. Mao, R. Du, Y. Wang, *Chem. Eng. J.* **2022**, 442, 136264.

[22] Y. Xu, M. Liu, S. Wang, K. Ren, M. Wang, Z. Wang, X. Li, L. Wang, H. Wang, *Appl. Catal. B-Environ.* **2021**, 298, 120493.

[23] W.-J. Liu, Z. Xu, D. Zhao, X.-Q. Pan, H.-C. Li, X. Hu, Z.-Y. Fan, W.-K. Wang, G.-H. Zhao, S. Jin, G. W. Huber, H.-Q. Yu, *Nat. Commun.* **2020**, 11, 265.

[24] G. Yang, Y. Jiao, H. Yan, Y. Xie, A. Wu, X. Dong, D. Guo, C. Tian, H. Fu, *Adv. Mater.* **2020**, 32, 2000455.

[25] L. Gao, Z. Liu, J. Ma, L. Zhong, Z. Song, J. Xu, S. Gan, D. Han, L. Niu, *Appl. Catal. B-Environ.* **2020**, 261, 118235.

[26] X. Deng, X. Kang, M. Li, K. Xiang, C. Wang, Z. Guo, J. Zhang, X.-Z. Fu, J.-L. Luo, *J. Mater. Chem. A* **2020**, 8, 1138.
